# Education Levels and Poststroke Cognitive Trajectories

**DOI:** 10.1001/jamanetworkopen.2025.2002

**Published:** 2025-03-26

**Authors:** Mellanie V. Springer, Rachael T. Whitney, Wen Ye, Emily M. Briceño, Alden L. Gross, Hugo J. Aparicio, Alexa S. Beiser, James F. Burke, Mitchell S. V. Elkind, Rebecca A. Ferber, Bruno Giordani, Rebecca F. Gottesman, Rodney A. Hayward, Virginia J. Howard, Adam S. Kollipara, Silvia Koton, Ronald M. Lazar, W. T. Longstreth, Sarah T. Pendlebury, Jeremy B. Sussman, Evan L. Thacker, Deborah A. Levine

**Affiliations:** 1 Department of Neurology and Stroke Program, University of Michigan, Ann Arbor; 2 Institute for Healthcare Policy and Innovation, University of Michigan, Ann Arbor; 3 Department of Internal Medicine, University of Michigan, Ann Arbor; 4 Cognitive Health Services Research Program, University of Michigan, Ann Arbor; 5 Department of Biostatistics, University of Michigan School of Public Health, Ann Arbor; 6 Department of Physical Medicine and Rehabilitation, University of Michigan, Ann Arbor; 7 Department of Epidemiology, Johns Hopkins Bloomberg School of Public Health, Baltimore, Maryland; 8 Department of Neurology, Boston University Chobanian & Avedisian School of Medicine, Boston, Massachusetts; 9 Framingham Heart Study, National Heart, Lung, and Blood Institute, Framingham, Massachusetts; 10 Department of Biostatistics, Boston University School of Public Health, Boston, Massachusetts; 11 Department of Neurology, Ohio State University College of Medicine, Columbus; 12 Department of Neurology, Vagelos College of Physicians and Surgeons , Columbia University, New York, New York; 13 Department of Epidemiology, Mailman School of Public Health, Columbia University, New York, New York; 14 Department of Psychiatry, Michigan Alzheimer’s Disease Center, University of Michigan, Ann Arbor; 15 Stroke Branch, National Institute of Neurological Disorders and Stroke, Bethesda, Maryland; 16 VA Ann Arbor Healthcare System, Ann Arbor, Michigan; 17 Department of Epidemiology, University of Alabama at Birmingham School of Public Health; 18 Department of Nursing, The Stanley Steyer School of Health Professions, Tel Aviv University, Tel Aviv, Israel; 19 Department of Neurology, Evelyn F. McKnight Brain Institute, Heersink School of Medicine, University of Alabama at Birmingham; 20 Department of Neurology, University of Washington, Seattle; 21 Department of Epidemiology, University of Washington, Seattle; 22 Wolfson Centre for Prevention of Stroke and Dementia, Nuffield Department of Clinical Neurosciences, University of Oxford, Oxford, United Kingdom; 23 Department of Acute General Medicine, National Institute for Health and Care Research Oxford Biomedical Research Centre, Oxford University Hospitals National Health Service Foundation Trust, Oxford, United Kingdom; 24 Department of Geratology, National Institute for Health and Care Research Oxford Biomedical Research Centre, Oxford University Hospitals National Health Service Foundation Trust, Oxford, United Kingdom; 25 Department of Public Health, Brigham Young University, Provo, Utah

## Abstract

**Importance:**

Acute stroke is associated with accelerated, years-long cognitive decline. Whether education levels are associated with faster cognitive decline after stroke is unclear.

**Objective:**

To evaluate the association of education level with poststroke cognitive decline and to determine whether age at stroke modifies the association.

**Design, Setting, and Participants:**

Individual participant data meta-analysis of 4 US cohort studies (January 1971 to December 2019). Analysis began August 2022 and was completed in January 2024.

**Exposures:**

Education level (less than high school, completed high school, some college, and college graduate).

**Main Outcomes and Measures:**

Harmonized cognitive outcomes were global cognition (primary outcome), memory, and executive function. Outcomes were standardized as *t* scores (mean [SD], 50 [10]); a 1-point difference represents a 0.1-SD difference in cognition, with higher score representing better function. Linear mixed-effect models estimated the trajectory of cognitive decline after incident stroke.

**Results:**

The analysis included 2019 initially dementia-free stroke survivors (1048 female [51.9%]; median [IQR] age at stroke, 74.8 [69.0-80.4] years; 339 with less than a high school education [16.7%]; 613 who completed high school [30.4%]; 484 with some college [24.0%]; 583 with a college degree or higher [28.9%]). Median (IQR) follow-up time after stroke was 4.1 (1.8-7.2) years. Compared with those with less than a high school degree, college graduates had higher initial poststroke performance in global cognition (1.09 points higher; 95% CI, 0.02 to 2.17 points higher), executive function (1.81 points higher; 95%CI, 0.38 to 3.24 points higher), and memory (0.99 points higher; 95% CI, 0.02 to 1.96 points higher). Compared with stroke survivors with less than a high school education, there was a faster decline in executive function among college graduates (−0.44 points/y faster; 95% CI, −0.69 to −0.18 points/y faster) and those with some college education(−0.30 points/y faster; 95% CI, −0.57 to −0.03 points/y faster). Education level was not associated with declines in global cognition or memory. Age did not modify the association of education with cognitive decline.

**Conclusions and Relevance:**

In this pooled cohort study, the trajectory of cognitive decline after stroke varied by education level and cognitive domain, suggesting that stroke survivors with a higher education level may have greater cognitive reserve but steeper decline in executive function than those with a lower education level.

## Key Points

**Question:**

Is level of education associated with trajectory of cognitive decline following a stroke?

**Findings:**

In this pooled cohort analysis of 2019 participants, stroke survivors who were college graduates and those with some college education had faster poststroke decline in executive function than stroke survivors who had less than a high school degree after adjusting for prestroke cognition. Education level was not associated with declines in global cognition or memory.

**Meaning:**

These findings suggest that high education level may be associated with faster decline in executive function in stroke survivors.

## Teaser

This cohort study analyzes associations of education level with global cognition, executive function, and memory among individuals with stroke.

## Introduction

Stroke increases dementia risk by as much as 50-fold.^1^ The association of education with poststroke dementia is unclear, but some studies have suggested that lower levels of education increase the risk for poststroke dementia.^2^ Previous studies evaluating the association of education with poststroke cognition examined cognitive impairment at a single time point after stroke or did not account for prestroke cognition.^2,3^ Because acute stroke is known to be associated with accelerated years-long cognitive decline,^4^ it is important to evaluate if education is associated with poststroke cognitive decline. Educational attainment has been considered a proxy for cognitive reserve, allowing the brain to preserve cognitive function despite brain injury occurring over the life course. Although it is known that older age increases the risk for poststroke cognitive decline,^5^ it is unclear whether age modifies the association of education with poststroke cognitive decline. Prior studies were restricted to patients with stroke who received a diagnosis of cognitive impairment at the time of stroke and did not control for prestroke cognition.^6^ Identifying which patients with stroke are at the highest risk for cognitive decline will help target future interventions to slow cognitive decline. Our objective was to evaluate the association of education with poststroke cognitive decline in a pooled longitudinal cohort of stroke survivors without prevalent cognitive impairment and to evaluate whether age modifies the association. We hypothesized that having a higher level of education would be associated with slower cognitive decline after stroke.

## Methods

This cohort study was approved by the University of Michigan institutional review board. Individual cohorts received institutional review board approval from their governing institutions. Participants provided written informed consent. The study adheres to the Strengthening the Reporting of Observational Studies in Epidemiology (STROBE) reporting guideline for cohort studies.

### Study Design, Participants, and Measurements

The Effect of Vascular Risk Factors on Cognitive Trajectories After Stroke (STROKE COG) study pooled individual participant data from 4 prospective cohort studies following reporting guidelines^7^ for individual participant data meta-analyses: the Atherosclerosis Risk in Communities Study (ARIC),^8^ Framingham Offspring Study (FOS),^9^ Reasons for Geographic and Racial Differences in Stroke Study (REGARDS),^10^ and Cardiovascular Health Study (CHS).^11^ The pooled cohort includes data from cohort baseline visits from January 1971 through December 2019. Derivation of the pooled cohort is detailed previously.^12^

This analysis included STROKE COG participants who had acute ischemic or hemorrhagic stroke during cohort follow-up, were 18 years of age or older at the time of acute stroke, had 1 or more cognitive assessments before acute stroke, and had 1 or more cognitive assessments after acute stroke. We excluded participants with cohort-defined dementia at or before the acute stroke, who were missing data on education level, or who self-reported a race other than Black or White, given the small number of other race categories and differences in cohort design. We included participants with a self-reported history of stroke prior to the acute stroke during follow-up because we have found no difference in the association of acute stroke during follow-up with cognitive decline when including or excluding participants with a self-reported history of stroke.^12^

### Cognitive Function Outcomes

Cognitive assessments were administered by trained cohort staff either in person (ARIC, CHS, and FOS)^8,9,11^ or by telephone (REGARDS)^10^ according to standardized protocols. Global cognition, executive function, and memory can be measured reliably and validly by telephone.^13^

Confirmatory factor analysis methods were used to statistically cocalibrate, or harmonize, individual test items across cohorts into the domains of global cognition, memory, and executive function.^14,15,16^ Additional details about the harmonization of cognitive tests are in the eMethods in Supplement 1. We obtained factor scores using regression-based methods in Mplus version 8,^17,18^ and standardized them to a *t* score metric (mean [SD], 50 [10]) at a participant’s first poststroke cognitive assessment. A 1-point difference equals a 0.1-SD difference in the distribution of cognition across the cohorts. Higher cognitive scores denote better cognitive performance. The primary outcome was global cognition. The secondary outcomes were memory and executive function.

### Measurement of Education

Cohort participants self-reported years of education, categorized as less than a high school degree, completed high school, some college but no degree, and college graduate or more. Cohort-specific details about the measurement of education can be found in the eMethods in Supplement 1.

### Covariates

We selected covariates based on their known association with cognitive decline and availability in the pooled cohort. Age (years) was measured at the time of acute stroke. Demographics were self-reported sex, self-reported race (Black or White), and cohort; race was included to account for known racial differences in cognition after stroke. Prestroke mean glucose, systolic blood pressure, body mass index (calculated as weight in kilograms divided by height in meters squared), estimated glomerular filtration rate, and low-density lipoprotein cholesterol were the arithmetic mean of all measurements before stroke. Income, current cigarette smoking, physical activity, history of myocardial infarction, and history of atrial fibrillation were the values nearest to and before acute stroke. Stroke type was ischemic or hemorrhagic. Prestroke cognition was the arithmetic mean of all global cognition, memory, or executive function scores before acute stroke. Cognitive testing was available for participants before stroke because repeated cognitive assessments were performed during cohort follow-up by study design. Depressive symptoms were measured using the Center for Epidemiologic Studies Depression Scale (CES-D)^19^ and summarized as the arithmetic mean of all scores after stroke due to cohort design. Higher scores indicate more depressive symptoms. Depression scores were available for a subset of participants (1134 participants for global cognition) and analyzed in a sensitivity analysis. The number of apolipoprotein E (ApoE) ε4 alleles was available for a subset of participants (1406 participants for global cognition) and analyzed in a sensitivity analysis. The eMethods in Supplement 1 describes covariate details.

### Statistical Analysis

#### Primary Analysis

We included participants with complete covariate data. Linear mixed-effects models were used to estimate longitudinal change in each continuous cognitive outcome as a function of education level, follow-up time, and the interaction between time and each education level to reflect associations of each level with cognitive change. The reference group was less than a high school degree. Model 1 included follow-up time, indicators for education level, an interaction between follow-up time and each education level, prestroke cognition, and participant-specific random effects for intercept and slope. Follow-up time was expressed as years since acute stroke and treated as a continuous measure. Model 2 further adjusted for cohort, age (at stroke), a 2-way interaction term between follow-up time and age, sex, a 2-way interaction term between follow-up time and sex,^12^ race, income, prestroke mean systolic blood pressure, prestroke mean glucose, prestroke mean low-density lipoprotein cholesterol, body mass index, smoking status, physical activity, history of myocardial infarction, history of atrial fibrillation, estimated glomerular filtration rate, stroke type, and a 3-way interaction term between follow-up time, age, and education. If there were no significant 3-way interactions, we reran the model, excluding the 3- way interaction. We evaluated model diagnostics to confirm model assumptions were met and results were not affected by influential points or outliers. We plotted cognitive trajectories for individual participants by education category using a spaghetti plot in order to visualize whether there were influential points or outliers. The threshold for significance was a 2-tailed *P* < .05. Analyses were performed using Stata version 18.0 (StataCorp LLC) from August 2022 to January 2024.

#### Sensitivity Analyses

To evaluate the robustness of our findings, we repeated model 2 (1) within each cohort (excluding FOS due to insufficient sample size), (2) restricting to participants with no history of stroke at cohort baseline, (3) including poststroke CES-D score and an interaction between follow-up time and poststroke CES-D score in participants with data on depressive symptoms, (4) including ApoE genotype and an interaction between follow-up time and ApoE genotype in the subset of participants with available genetic data, (5) censoring observations at the time of second stroke during cohort follow-up, (6) excluding participants with the lowest 5% of cognitive scores before stroke to exclude a floor effect among those with the poorest prestroke cognitive function, and (7) performing a joint modeling analysis to account for any observations that were missing not at random. Joint models address data that are missing not at random by jointly monitoring longitudinal data and by considering the pattern of missing data. Our joint model is of longitudinal and survival data linked together through shared random effects. The longitudinal model is a linear mixed-effects model, as in our primary analysis. The survival model is a Weibull survival analysis, with the outcome of median survival time. We chose the median survival time as the outcome because drop-out due to death is likely associated with both education level and cognitive trajectory. Participants were censored at death, end of follow-up, and loss to follow-up. The same covariates (as in the primary analysis) were used in both models, with the exception of time interactions being removed from the survival model.

## Results

The final sample included 2019 initially dementia-free participants (1239 White [61.4%]; 780 Black [38.6%]; 1048 female [51.9%]) with acute stroke (1876 ischemic and 143 hemorrhagic) during cohort follow-up (Figure). The median (IQR) age at acute stroke was 74.8 (69.0-80.4) years. The median (IQR) follow-up time after stroke was 4.1 (1.8-7.2) years. There were 138 participants who had a history of stroke before the stroke that occurred during cohort follow-up. There were 339 participants (16.7%) with less than a high school education, 613 (30.4%) who completed high school, 484 (24.0%) with some college but no degree, and 583 (28.9%) with a college degree or more.

**Figure.**
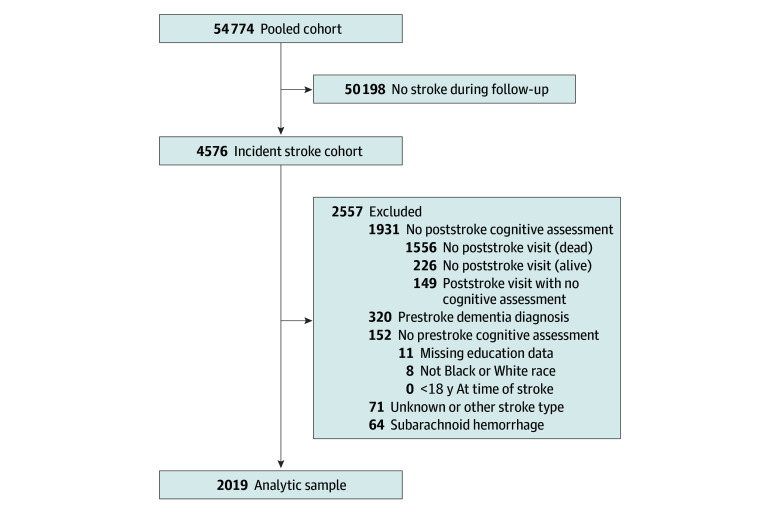
Participant Flow Diagram

Table 1 shows participant characteristics by education category. Of the 583 participants who graduated from college, 332 (56.9%) were male and 283 (48.5%) were Black. Of the 339 participants with less than a high school degree, 156 (46.0%) were male and 102 (30.1%) were Black. Participants with less than a high school degree had higher prestroke mean systolic blood pressure, glucose, and low-density lipoprotein cholesterol values than participants in higher education categories. Participants in each education category completed a median of 2 or more cognitive assessments after stroke and a median of 2 or more cognitive assessments before stroke (Table 1). There were no points or outliers influencing the results (eFigure in Supplement 1).

**Table 1.** Characteristics of Participants at First Poststroke Cognitive Assessment by Education Category

Characteristic	Participants by education category, No (%) (N = 2019)				
	Less than high school degree (n = 339)	Completed high school (n = 613)	Some college but no degree (n = 484)	College graduate or more (n = 583)	*P* value^a^
Age at time of stroke, y					
Median (IQR)	75.7 (70.6-81.0)	74.4 (68.6-80.2)	74.7 (68.9-80.8)	74.7 (69.0-79.9)	.20
Range	52.9-94.9	44.1-93.1	48.8-95.8	46.6-96.4	
<65	40 (11.8)	86 (14.0)	70 (14.5)	86 (14.8)	.63
≥65	299 (88.2)	527 (86.0)	414 (85.5)	497 (85.2)	
Acute stroke type					
Hemorrhagic	17 (5.0)	48 (7.8)	32 (6.6)	46 (7.9)	.33
Ischemic	322 (95.0)	565 (92.2)	452 (93.4)	537 (92.1)	
Sex					
Male	156 (46.0)	255 (41.6)	228 (47.1)	332 (56.9)	<.001
Female	183 (54.0)	358 (58.4)	256 (52.9)	251 (43.1)	
Race					
Black	102 (30.1)	198 (32.3)	197 (40.7)	283 (48.5)	<.001
White	237 (69.9)	415 (67.7)	287 (59.3)	300 (51.5)	
Cohort					
ARIC	55 (16.2)	97 (15.8)	31 (6.4)	99 (17.0)	<.001
CHS	123 (36.3)	128 (20.9)	101 (20.9)	81 (13.9)	
FOS	10 (2.9)	38 (6.2)	48 (9.9)	39 (6.7)	
REGARDS	151 (44.5)	350 (57.1)	304 (62.8)	364 (62.4)	
Income, $					
<5000	14 (4.1)	15 (2.4)	5 (1.0)	4 (0.7)	<.001
5000-24 999	184 (54.3)	243 (39.6)	126 (26.0)	76 (13.0)	
25 000-34 999	37 (10.9)	100 (16.3)	98 (20.2)	81 (13.9)	
35 000-49 999	21 (6.2)	86 (14.0)	82 (16.9)	106 (18.2)	
≥50 000	11 (3.2)	67 (10.9)	101 (20.9)	241 (41.3)	
Refused or missing	72 (21.2)	102 (16.6)	72 (14.9)	75 (12.9)	
Current cigarette smoking	45 (13.3)	82 (13.4)	63 (13.0)	63 (10.8)	.53
Physical activity	216 (64.1)	400 (66.2)	320 (66.4)	418 (72.1)	.05
Body mass index, median (IQR)^b^	27.9 (24.8-31.6)	27.5 (24.7-31.7)	27.6 (24.9-31.4)	27.3 (25.0-30.7)	.50
Waist circumference, median (IQR), cm	98.2 (90.5-107.2)	97.2 (88.9-106.7)	96.5 (88.9-106.0)	97.3 (89.1-105.3)	.39
No. of alcoholic drinks per week, median (IQR)	1.0 (1.0-1.0)	1.0 (1.0-2.0)	1.0 (1.0-2.0)	1.0 (1.0-2.0)	<.001
Prestroke cumulative SBP, mean (SD), mmHg	142.2 (18.9)	138.8 (19.5)	135.3 (17.5)	135.3 (17.6)	<.001
Prestroke cumulative fasting glucose, mean (SD), mg/dL	122.5 (54.3)	114.8 (43.7)	110.2 (42.7)	106.9 (36.1)	<.001
Prestroke cumulative LDL-C, mean (SD), mg/dL	127.3 (35.7)	123.6 (37.4)	119.0 (35.5)	119.4 (33.8)	.002
Glomerular filtration rate, median (IQR), mL/min	71.8 (54.8-89.0)	74.8 (57.9-90.9)	72.8 (58.0-89.4)	75.1 (60.6-89.6)	.34
History of acute myocardial infarction	51 (15.4)	96 (15.9)	67 (13.9)	80 (13.9)	.73
History of atrial fibrillation	25 (7.5)	46 (7.6)	36 (7.6)	51 (8.9)	.81
No. of ApoE ε4 alleles					
0	185 (54.6)	311 (50.7)	236 (48.8)	272 (46.7)	.05
1	67 (19.8)	105 (17.1)	94 (19.4)	101 (17.3)	
2	6 (1.8)	6 (1.0)	13 (2.7)	10 (1.7)	
Missing	81 (23.9)	191 (31.2)	141 (29.1)	200 (34.3)	
Follow-up time after stroke for global cognition, median (IQR), y	4.2 (1.7-6.8)	3.6 (1.7-6.7)	4.2 (1.8-7.4)	4.3 (2.0-8.0)	.04
Prestroke cumulative mean cognitive scores, median (IQR)					
Global cognition	47.9 (43.0-52.5)	52.7 (49.3-56.1)	53.7 (49.9-57.1)	55.0 (51.7-58.1)	<.001
Executive function	42.8 (36.5-46.8)	49.4 (43.6-54.1)	50.2 (45.1-55.5)	53.5 (48.0-57.5)	<.001
Memory	51.2 (49.1-55.3)	54.3 (50.7-56.4)	54.6 (51.6-57.4)	55.0 (52.9-57.4)	<.001
No. of prestroke cognitive assessments, median (IQR)					
Global cognition	3.0 (2.0-7.0)	4.0 (2.0-9.0)	5.0 (3.0-9.0)	5.0 (2.0-10.0)	<.001
Executive function	2.0 (1.0-4.0)	2.0 (1.0-4.0)	2.0 (1.0-4.0)	2.0 (1.0-4.0)	.58
Memory	3.0 (2.0-6.0)	4.0 (2.0-7.0)	4.0 (2.0-7.0)	4.0 (2.0-7.0)	<.001
Cognitive scores at first poststroke cognition assessment, median (IQR)					
Global cognition	43.2 (35.8-52.0)	49.9 (41.3-57.1)	51.8 (44.5-58.3)	52.0 (44.4-58.5)	<.001
Executive function	39.8 (33.9-46.1)	44.2 (37.5-50.6)	46.8 (39.8-53.3)	48.4 (41.6-54.8)	<.001
Memory	49.1 (43.5-57.4)	50.2 (49.1-57.4)	55.0 (49.1-57.4)	57.4 (49.1-57.4)	<.001
No. of poststroke cognitive assessments, median (IQR)					
Global cognition	3.0 (1.0-6.0)	3.0 (2.0-6.0)	4.0 (2.0-8.0)	4.0 (2.0-8.0)	<.001
Executive function	2.0 (1.0-3.0)	2.0 (1.0-3.0)	2.0 (1.0-3.0)	2.0 (1.0-3.0)	.21
Memory	2.0 (1.0-5.0)	2.0 (1.0-4.0)	3.0 (1.0-6.0)	3.0 (1.0-5.0)	<.001
Died during follow-up	220 (64.9)	316 (51.5)	217 (44.8)	231 (39.6)	<.001

Abbreviations: ApoE, apolipoprotein E; ARIC, Atherosclerosis Risk in Communities Study; CHS, Cardiovascular Health Study; FOS, Framingham Offspring Study; LDL-C, low-density lipoprotein cholesterol; REGARDS, Reasons for Geographic and Racial Differences in Stroke Study; SBP, systolic blood pressure.

SI conversion factor: To convert fasting glucose to millimoles per liter, multiply by 0.0555; to convert LDL-C to millimoles per liter, multiply by 0.0259.

^a^ Differences between continuous variables were assessed using Kruskal-Wallis or analysis of variance tests and differences between categorical variables were assessed using χ^2^ tests.

^b^ Body mass index was calculated as weight in kilograms divided by height in meters squared.

### Education and Global Cognitive Performance After Stroke

On average, college graduates had higher mean initial poststroke performance in global cognition than survivors with less than a high school education (1.09 points higher; 95% CI, 0.02 to 2.17 points higher). Survivors with a high school degree or some college education, on average, had initial poststroke global cognition performance that did not significantly differ from survivors with less than a high school degree (Table 2, model 1).

**Table 2.** Association of Education With Poststroke Cognitive Decline

Model	Global cognition		Executive function		Memory	
	Estimate (95% CI)^a^	*P* value	Estimate (95% CI)	*P* value	Estimate (95% CI)	*P* value
Model 1: no adjustment^b^						
Participants, total No.	2019		1451		1805	NA
Initial mean poststroke cognitive score in those with less than high school education	46.73 (45.91 to 47.56)	<.001	45.56 (44.45 to 46.67)	<.001	49.11 (48.34 to 49.88)	<.001
Difference in initial poststroke cognitive score by education, points (reference: less than high school degree)	NA		NA		NA	
High school	0.21 (−0.83 to 1.25)	.12	−0.46 (−1.83 to 0.90)	<.001	0.32 (−0.63 to 1.27)	.16
Some college	0.54 (−0.54 to 1.62)		1.44 (0.01 to 2.86)		0.34 (−0.65 to 1.32)	
College or more	1.09 (0.02 to 2.17)		1.81 (0.38 to 3.24)		0.99 (0.02 to 1.96)	
Poststroke cognitive slope in those with less than high school degree, points/y	−0.62 (−0.79 to −0.46)	<.001	−0.34 (−0.54 to −0.14)	<.001	−0.28 (−0.45 to −0.12)	<.001
Difference in poststroke cognitive slope by education, points/y						
High school vs less than high school	0.13 (−0.07 to 0.34)	.10	−0.04 (−0.29 to 0.21)	.004	−0.12 (−0.32 to 0.09)	<.001
Some college vs less than high school	0.25 (0.04 to 0.45)		−0.28 (−0.54 to −0.01)		0.18 (−0.03 to 0.39)	
College or more vs less than high school	0.09 (−0.11 to 0.29)		−0.37 (−0.63 to −0.12)		−0.14 (−0.34 to 0.06)	
Model 2: Full adjustment^c^						
Participants, total No.	1890		1394		1684	NA
Initial mean poststroke cognitive score in those with less than high school degree	47.79 (44.56 to 51.03)	<.001	43.73 (39.78 to 47.69)	<.001	50.16 (47.30 to 53.03)	<.001
Difference in initial poststroke cognitive score by education, points (reference: less than high school degree)						
High school	0.05 (−1.01 to 1.12)	.61	−0.13 (−1.50 to 1.25)	.01	0.28 (−0.68 to 1.24)	.02
Some college	0.05 (−1.07 to 1.17)		0.93 (−0.52 to 2.39)		−0.12 (−1.12 to 0.89)	
College or more	0.57 (−0.58 to 1.72)		1.84 (0.34 to 3.35)		1.09 (0.06 to 2.11)	
Difference in initial poststroke cognitive score by age, points (per 10-y increase)	−1.34 (−1.78 to −0.90)	<.001	−1.41 (−2.00 to −0.81)	<.001	−1.06 (−1.46 to −0.67)	<.001
Difference in initial poststroke cognitive score for female sex, points	0.50 (−0.19 to 1.19)	.15	0.52 (−0.40 to 1.44)	.26	0.55 (−0.07 to 1.17)	.08
Poststroke cognitive slope in those with less than high school degree, points/y	−0.35 (−0.53 to −0.16)	<.001	−0.14 (−0.38 to 0.09)	.23	−0.11 (−0.30 to 0.07)	.23
Difference in poststroke cognitive slope by education, points/y, (reference: less than high school degree)						
High school	0.11 (−0.09 to 0.31)	.09	−0.08 (−0.34 to 0.17)	.001	−0.08 (−0.29 to 0.13)	.001
Some college	0.21 (0.00 to 0.41)		−0.30 (−0.57 to −0.03)		0.18 (−0.03 to 0.39)	
College or more	0.01 (−0.19 to 0.21)		−0.44 (−0.69 to −0.18)		−0.14 (−0.34 to 0.06)	
Difference in poststroke cognitive slope by age, points (per 10-y increase)	−0.23 (−0.30 to −0.15)	<.001	−0.11 (−0.21 to −0.01)	.03	−0.07 (−0.14 to 0.01)	.09
Difference in poststroke cognitive slope for female sex, points/y	−0.13 (−0.26 to −0.00)	.045	−0.11 (−0.29 to 0.06)	.18	−0.12 (−0.25 to 0.01)	.06

Abbreviation: NA, not applicable.

^a^ All cognitive measurements are set to a *t* score metric (mean [SD], 50 [10]). A 1-point difference represents a 0.1-SD difference in the distribution of cognition across the 4 cohorts. Higher cognitive scores indicate better performance.

^b^ Model 1 includes follow-up time, education, education × follow-up time, and prestroke cognition.

^c^ Model 2 added cohort, age, age × follow-up time, sex, sex × follow-up time, race, income, prestroke mean systolic blood pressure, prestroke mean fasting glucose, prestroke mean low-density lipoprotein cholesterol, body mass index, smoking status, physical activity, history of myocardial infarction, history of atrial fibrillation, estimated glomerular filtration rate, and stroke type to model 1.

Among survivors with less than a high school degree, global cognition declined significantly after stroke before (−0.62 points/y; 95% CI, −0.79 to −0.46 points/y; *P* < .001) and after adjustment (−0.35 points/y; 95% CI, −0.53 to −0.16 points/y; *P* < .001) (Table 2, models 1 and 2). Survivors with some college education had a slower rate of decline in global cognition after stroke than survivors with less than a high school degree (0.25 points/y slower; 95% CI, 0.04 to 0.45 points/y slower) (Table 2, model 1); however, there was no longer an association after adjustment (Table 2, model 2).

### Education and Executive Function After Stroke

On average, compared with survivors with less than a high school degree, survivors with some college education had initial poststroke executive function scores that were 1.44 (95% CI, 0.01 to 2.86) points higher, and college graduates had initial scores that were 1.81 (95% CI, 0.38 to 3.24) points higher (Table 2, model 1). Among survivors with less than a high school degree, executive function declined significantly after stroke (−0.34 points/y; 95% CI, −0.54 to −0.14 points/y; *P* < .001), but there was no longer an association after adjustment (Table 2, models 1 and 2). Survivors with some college education had a faster decline in executive function after stroke compared with survivors with less than a high school degree (before adjustment, −0.28 points/y faster; 95% CI, −0.54 to −0.01 points/y faster; after adjustment, −0.30 points/y faster; 95% CI, −0.57 to −0.03 points/y faster). College graduates had a faster decline in executive function after stroke compared with those with less than a high school degree (before adjustment, −0.37 points/y faster; 95% CI, −0.63 to −0.12 points/y faster; after adjustment, −0.44 points/y faster; 95% CI, −0.69 to −0.18 points/y faster). There was no difference in the rate of decline in executive function after stroke between survivors with a high school degree and survivors with less than a high school degree (Table 2, model 2).

### Education and Memory After Stroke

College graduates had higher initial poststroke memory scores than survivors with less than a high school degree (0.99 points higher; 95% CI, 0.02 to 1.96 points higher) (Table 2, model 1). Among survivors with less than a high school degree, memory declined significantly after stroke before (−0.28 points/y; 95% CI, −0.45 to −0.12 points/y; *P* < .001) but not after adjustment (−0.11 points/y; 95% CI, −0.30 to 0.07 points/y; *P* = .23) (Table 2, models 1 and 2). Survivors with a high school degree did not differ in the rate of change in memory after stroke compared with those with less than a high school degree (Table 2, model 2). Pairwise comparisons showed that survivors with some college education had a slower decline in memory after stroke than college graduates (0.32 points/y; 95% CI, 0.15 to 0.48 points/y).

### Age and the Association of Education With Cognitive Decline After Stroke

Age at acute stroke did not modify the association of education level with global cognition after stroke (overall *P* = .66) (eTable 1 in Supplement 1). Age at acute stroke did not modify the association of education level with executive function after stroke (overall *P* = .18) (eTable 1 in Supplement 1). Age at acute stroke did not modify the association of education level with memory after stroke (overall *P* = .16) (eTable 1 in Supplement 1).

### Sensitivity Analyses

Results were consistent among the subset of participants with data on the ApoE ε4 allele and adjusted for ApoE ε4 status. The magnitude of the association of education level with decline in executive function in the sensitivity analysis was similar to the main analysis, although the overall *P* value was .047 compared with an overall *P* value of .001 in the main analysis (eTable 2 in Supplement 1). Results were consistent when excluding participants with a history of stroke at cohort baseline. The magnitude of the association of education level with decline in executive function was almost identical in the sensitivity analysis compared with the main analysis (eTable 3 in Supplement 1).

Results were consistent when adjusting for depression scores. The magnitude of the associations of education level with decline in executive function were attenuated in the sensitivity analysis but remained statistically significant at an overall *P* value of .047 (eTable 4 in Supplement 1). Results were consistent and of similar magnitude as the primary analysis when censoring participants at recurrent stroke; consistent with the primary analysis, there was faster decline in executive function among the college-educated. There was no association of some college education with decline in executive function when adjusting for depression scores (eTable 4 and eTable 5 in Supplement 1). Findings were consistent in the 3 individual cohort analyses, but some contrasts were no longer statistically significant due to smaller sample sizes (eTables 6-8 in Supplement 1).

Results were consistent when excluding participants with the lowest 5% of cognitive scores before stroke (eTable 9 in Supplement 1). Specifically, the magnitude of the association of education level with decline in executive function after stroke for each education category was similar in the sensitivity analysis to that in the main analysis. The association of college education with memory after stroke was also of the same magnitude as in the main analysis.

Results of our joint model showed that there was no association of higher education level with faster decline in executive function after stroke. Participants with some college education had a slower decline in memory after stroke than college graduates, as was found in the main analysis (eTable 10 in Supplement 1). For all the aforementioned results, model diagnostics showed no violation of model assumptions or influential points that could have biased the results.

## Discussion

In this pooled cohort study, we found that college graduates had higher mean initial poststroke performance in global cognition but there was no association of education level with the trajectory of global cognition after stroke. Participants who had some college education and those who graduated college had faster decline in executive function after stroke compared with those with less than a high school degree. Participants with some college education had a slower decline in memory after stroke than college graduates. Age at time of stroke did not modify any of the education-cognition associations.

Our finding that attending college was associated with higher initial poststroke executive function scores but faster decline over time is similar to previous findings in stroke-free adults. Although we hypothesized that those with higher education would have slower cognitive decline after stroke, a plausible explanation exists for the opposite finding. Studies have shown an association of higher education with higher cognitive ability,^20^ but age-related brain atrophy or pathology continues to occur over time regardless of education level.^21^ Higher education may enable compensation for brain changes,^22^ thereby preserving higher cognitive ability until a critical threshold of brain injury is reached at which compensation fails and rapid cognitive decline occurs even after adjusting for differences in cognitive ability prior to the brain injury.^23,24^ Potential mechanisms of cognitive compensation among more highly educated older adults include greater recruitment of brain regions important for cognition or recruitment of alternative neural networks compared with their less-educated peers.^25,26^ Similar to the faster decline in executive function seen in the more highly educated after a dementia diagnosis,^24^ stroke is an acute injury to the brain that surpasses the threshold of brain injury at which executive function can be maintained in college-educated adults.

Participants with some college education had a slower decline in memory after stroke compared with college graduates. Previous studies have shown a decline in memory acutely after stroke, but no change in long-term memory trajectories after stroke compared with before stroke.^4,27^ We extend these findings by showing that memory trajectories after stroke varied with education level, with faster rates of decline in college graduates than in those who attended but did not graduate college. These findings are in line with previous reports on individuals with incident Alzheimer disease, showing that each additional year of education is associated with a faster rate of cognitive decline.^24^ It is possible that the college graduates have more advanced brain pathology but are functioning at a higher level than their less-educated peers. Previous research has shown that education no longer has a protective effect in the setting of high levels of brain degeneration.^28^ The accelerated memory decline that we observed in older adults who graduated college as compared with those with some college experience might reflect that their age-related brain changes in combination with the acute stroke lesion exceed the threshold of brain injury at which cognitive compensation can occur.

The presence of ApoE ε4 and recurrent stroke did not appear to alter the association of education level with cognitive decline. A higher number of ApoE ε4 alleles, a genetic risk factor for Alzheimer disease, has been associated with faster cognitive decline after stroke.^12^ Higher risk for cognitive impairment has been shown after recurrent strokes than after a single stroke.^29^ We observed that regardless of the number of ApoE ε4 alleles or number of strokes, college-educated stroke survivors had faster decline in executive function. This finding suggests that the critical threshold of brain injury above which cognitive compensation fails in the highly educated does not depend on underlying genetic risk and can be reached after a single stroke.

### Strengths and Limitations

Strengths of the study include inclusion of a large sample of participants with stroke who had cognitive measures prior to stroke and repeated cognitive measures over time after stroke, enabling the characterization of poststroke cognitive trajectories that account for prestroke cognition. Among limitations, we did not have information about how the degree of brain atrophy, severity of white matter ischemic disease, or stroke severity compared among stroke participants with different education levels. Second, our study population was limited to cohort participants who self-reported Black or White race. Given that social determinants of health (eg, access to health care, education quality, and neighborhood environment) impact cognition and may be associated with the social construct of race,^30,31,32^ our findings may not be generalizable to other racial or ethnic groups. Third, given that we did not observe a decline in memory after stroke among participants with the least amount of education (ie, participants with less than a high school education), our finding of faster decline in memory among the college-educated compared with those with some college education might have been due to type 1 error. Fourth, we had insufficient information to evaluate the contribution of prestroke occupation to the observed association of education with cognitive decline. Fifth, cohort visits occurred at fixed intervals and timing of stroke relative to cohort visits was variable. Given that years may have passed between consecutive visits in some cohorts (eg, ARIC), participants varied in the time between stroke and their cognitive assessment. However, linear mixed-effects models handle varying lengths of time between measurements by incorporating a time variable as a fixed effect, allowing the model to account for the different time points without requiring equally spaced intervals; the random-effects component then accounts for the variability between individuals, effectively adjusting for the different timing of measurements within each participant. Given that the variability was random and accounted for in the linear mixed-effects models, it is not expected to have influenced our observed associations of education with poststroke cognition. Sixth, more cognitively impaired participants with stroke might have been less likely to complete follow-up cohort visits. It is also possible that participants with stroke in our cohort with less than a high school education had better cognitive performance than those who dropped out of study follow-up. Furthermore, it has been shown that some participants who self-report a low education level may have actually attained more years of education.^33^ Therefore, we may have underestimated the association of education level with cognitive decline. Further studies could be performed to assess the replicability of our findings.

## Conclusions

The findings of this cohort study suggest that higher education level may be associated with faster cognitive decline after stroke. In designing interventions to slow cognitive decline after stroke, researchers should consider evaluating whether the efficacy of such interventions vary by education level.

## Notes

Supplement 1.
eMethods.
**eFigure.** The Association Between Executive Function Score and Time From Stroke **eTable 1.** Association of Education and Post-Stroke Cognitive Decline, Including a Three-Way Interaction Between Age, Education and Time **eTable 2.** Association of Education and Post-Stroke Cognitive Decline Including Adjustment for ApoE Genotype Among Those With Genetic Data **eTable 3.** Association of Education and Post-Stroke Cognitive Decline, in Participants With No History of Stroke at Baseline **eTable 4.** Association of Education and Post-Stroke Cognitive Decline Including Adjustment for Post-Stroke Depressive Symptoms **eTable 5.** Association of Education and Post-Stroke Decline in Global Cognitive Performance, Censoring Observations at Time of Second Incident Stroke **eTable 6.** Association of Education and Post-Stroke Cognitive Decline, in Participants of ARIC **eTable 7.** Association of Education and Post-Stroke Cognitive Decline, in Participants of CHS **eTable 8.** Association of Education and Post-Stroke Cognitive Decline, in Participants of REGARDS **eTable 9.** Association of Education and Post-Stroke Cognitive Decline in Which Participants With Pre-Stroke Cognitive Function in or Below the 5th Percentile Are Dropped **eTable 10.** Association of Education and Post-Stroke Cognitive Decline in a Joint Model of Longitudinal and Survival Data

Supplement 2.
Data Sharing Statement
