# Supplementary material for: Education Levels and Poststroke Cognitive Trajectories
Source: JAMA Netw Open. 2025 Mar 26;8(3):e252002. doi: 10.1001/jamanetworkopen.2025.2002 (PMC11947833; doi:10.1001/jamanetworkopen.2025.2002)
Supplement: Supplement 1. — eMethods. eFigure. The Association Between Executive Function Score and Time From Stroke eTable 1. Association of Education and Post-Stroke Cognitive Decline, Including a Three-Way Interaction Between Age, Education and Time eTable 2. Association of Education and Post-Stroke Cognitive Decline Including Adjustment for ApoE Genotype Among Those With Genetic Data eTable 3. Association of Education and Post-Stroke Cognitive Decline, in Participants With No History of Stroke at Baseline eTable 4. Association of Education and Post-Stroke Cognitive Decline Including Adjustment for Post-Stroke Depressive Symptoms eTable 5. Association of Education and Post-Stroke Decline in Global Cognitive Performance, Censoring Observations at Time of Second Incident Stroke eTable 6. Association of Education and Post-Stroke Cognitive Decline, in Participants of ARIC eTable 7. Association of Education and Post-Stroke Cognitive Decline, in Participants of CHS eTable 8. Association of Education and Post-Stroke Cognitive Decline, in Participants of REGARDS eTable 9. Association of Education and Post-Stroke Cognitive Decline in Which Participants With Pre-Stroke Cognitive Function in or Below the 5th Percentile Are Dropped eTable 10. Association of Education and Post-Stroke Cognitive Decline in a Joint Model of Longitudinal and Survival Data [file jamanetwopen-e252002-s001.pdf]

Supplemental Online Content

Springer MV, Whitney RT, Ye W, et al. Education levels and poststroke cognitive trajectories. *JAMA Netw. Open*. 2025;8(3):e252002. doi:10.1001/jamanetworkopen.2025.2002

eMethods.
eFigure. The Association Between Executive Function Score and Time From Stroke
eTable 1. Association of Education and Post-Stroke Cognitive Decline, Including a Three-Way Interaction Between Age, Education and Time
eTable 2. Association of Education and Post-Stroke Cognitive Decline Including Adjustment for ApoE Genotype Among Those With Genetic Data
eTable 3. Association of Education and Post-Stroke Cognitive Decline, in Participants With No History of Stroke at Baseline
eTable 4. Association of Education and Post-Stroke Cognitive Decline Including Adjustment for Post-Stroke Depressive Symptoms
eTable 5. Association of Education and Post-Stroke Decline in Global Cognitive Performance, Censoring Observations at Time of Second Incident Stroke
eTable 6. Association of Education and Post-Stroke Cognitive Decline, in Participants of ARIC
eTable 7. Association of Education and Post-Stroke Cognitive Decline, in Participants of CHS
eTable 8. Association of Education and Post-Stroke Cognitive Decline, in Participants of REGARDS
eTable 9. Association of Education and Post-Stroke Cognitive Decline in Which Participants With Pre-Stroke Cognitive Function in or Below the 5th Percentile Are Dropped
eTable 10. Association of Education and Post-Stroke Cognitive Decline in a Joint Model of Longitudinal and Survival Data

This supplemental material has been provided by the authors to give readers additional information about their work.

© 2025 Springer MV et al. *JAMA Network Open*.

eMethods

Measurement of Education- In the ARIC, CHS, and REGARDS cohorts, education was a categorical variable and collected at the baseline visit. In the FOS cohort, education was expressed as number of years and collected at visit 2.

Covariates- Pre-stroke glucose was converted to a fasting value for those participants who did not fast prior to their blood draw. Participants were asked to fast prior to blood draws at each visit, except that CHS participants were not asked to fast prior to blood draws at cohort evaluation year 7. Information on fasting status was collected at all visits. A correction factor of 1.8 mg/dL was applied to non-fasting glucose measurements based on prior research.[1] Findings were similar with and without the correction. Systolic blood pressure was measured in mmHg. Body mass index was calculated as weight in kilograms divided by height squared in meters. Glomerular filtration rate (mL/min/1.73m^2) was estimated using the Chronic Kidney Disease Epidemiology Collaboration equation. Low-density lipoprotein cholesterol is measured in the units mg/dL. Income was categorized as <$5,000, $5,000-$24,999, $25,000-$34,999, $35,000-$49,999, ≥$50,000, and missing/refused. Current cigarette smoking was a categorical variable (yes vs. no). Physical activity was categorized as any vs. none in the past 2- 4 weeks. The number of ApoE4 alleles was categorized as ≥1 allele vs 0 alleles.

Harmonization of cognitive measures- Methods based in item response theory were used to statistically co-calibrate summary factors for cognitive performance using items that were unique to a cohort and items common across cohorts. Confirmatory factor analysis models for global cognitive performance (primary outcome), memory, and executive function were estimated using the ARIC cohort (reference cohort). We then estimated models for other cohorts fixing item loadings and thresholds to their values in the reference cohort. Factors scores were generated from a final model that included all cohorts and no freely estimated parameters.

© 2025 Springer MV et al. *JAMA Network Open*.

Cognitive Tests by Domain

Global cognition domain:
Animal Naming (ARIC, CHS, FOS, REGARDS), Baddeley and Papagno Divided Attention Task (CHS), Block Design Test (FOS: WAIS; CHS: WAIS-R), Boston Naming Test (ARIC, CHS, FOS), California Verbal Learning Test (CHS), Delayed Word Recall test (ARIC), Consortium to Establish a Registry for Alzheimer's Disease (CERAD) Word List Learning (REGARDS), Delayed Word Recall Test (ARIC), Digit Span Test (ARIC: WMS-R; CHS: WAIS-R; FOS: WAIS), Digit Symbol Substitution Test/Digit Symbol Coding Test (ARIC: WAIS-R), Finger Tapping Test (FOS, ARIC), Grooved Pegboard Test (CHS), Hooper Visual Organization Test (FOS), Letter Fluency Test (ARIC, CHS, FOS, REGARDS), Logical Memory Test (ARIC: WMS-R; FOS:WMS), Mini-Mental State Examination (ARIC, CHS, FOS), Modified Mini-Mental State Examination (CHS), Montreal Cognitive Assessment (REGARDS), Paired Associates Learning (FOS: WMS), Ravens Colored Progressive Matrices (CHS), Rey-Osterrieth Complex Figure Test (CHS), Semantic Word Generation (CHS), Similarities (FOS: WAIS), Six Item Screener (REGARDS), Stroop Neuropsychological Screening Test (CHS), Telephone Interview for Cognitive Status (ARIC, CHS), Trail Making Test (ARIC, CHS, FOS), Visual Reproduction (FOS: WMS).

Memory domain:
California Verbal Learning Test (CHS), Consortium to Establish a Registry for Alzheimer's Disease (CERAD) Word List Learning (REGARDS), Delayed Word Recall test (ARIC), Digit Symbol Substitution Test (ARIC: Incidental Learning), Logical Memory Test (ARIC: WMS-R; FOS: WMS), Mini-Mental State Examination recall items (ARIC), Modified Mini-Mental State Examination delayed recall items (CHS), Telephone Interview for Cognitive Status recall items (ARIC, CHS), Montreal Cognitive Assessment recall items (REGARDS), Paired Associates Learning (FOS: WMS), Rey Complex Figure Test Recall (CHS), Visual Reproduction (FOS: WMS).

Executive function domain:
Animal Naming (ARIC, CHS, FOS, REGARDS), Baddeley and Papagno Divided Attention Task (CHS), Block Design Test (FOS: WAIS; CHS: WAIS-R), California Verbal Learning Test (CHS; semantic clustering), Digit Span Test (ARIC: WMS-R; CHS: WAIS-R; FOS: WAIS), Digit Symbol Substitution Test (ARIC, CHS: WAIS-R), Letter Fluency Test (ARIC, CHS, FOS, REGARDS), Mini-Mental State Examination subtraction/backward spelling items (ARIC; CHS; FOS); Modified Mini-Mental State Examination animal naming, counting, similarities items (ARIC); Ravens Colored Progressive Matrices (CHS), Similarities (FOS: WAIS), Stroop Neuropsychological Screening Test (CHS), Telephone Interview for Cognitive Status items counting and subtraction items (ARIC, CHS); Trail Making Test (ARIC, CHS, FOS).

Note: WAIS is Wechsler Adult Intelligence Scale. WAIS-R is Wechsler Adult Intelligence Scale – Revised. WMS is Wechsler Memory Scale. WMS-R is Wechsler Memory Scale – Revised.

Reference
1. Moebus S, Göres L, Lösch C, Jöckel KH. Impact of time since last caloric intake on blood glucose levels. *Eur J Epidemiol*. Sep 2011;26(9):719-28. doi:10.1007/s10654-011-9608-z

© 2025 Springer MV et al. *JAMA Network Open*.

eFigure. The association between executive function score and time from stroke

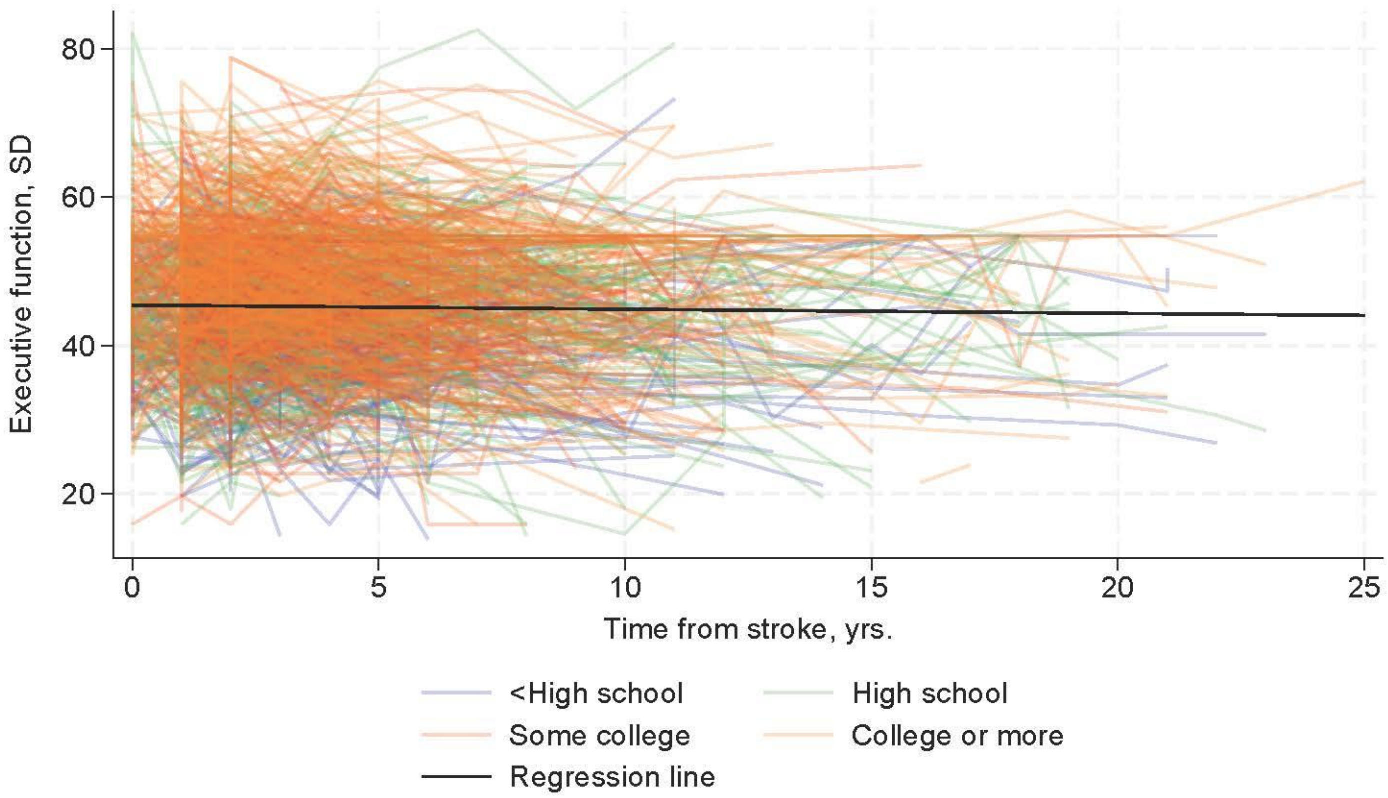

The spaghetti plot shows the association between executive function score and time after stroke for individual participants in each education category.

© 2025 Springer MV et al. *JAMA Network Open*.

eTable 1. Association of education and post-stroke cognitive decline, including a three-way interaction between age, education and time.

Coefficient	Global Cognition[a]		Executive Function		Memory	
Participants, total No.	1890		1394		1684	
Initial post-stroke cognitive score in those with less than high school education	47.46 (44.07,50.85)	<0.001	43.10 (38.90, 47.30)	<0.001	50.48 (47.46, 53.50)	<0.001
Difference in initial post-stroke cognitive score by education (Reference: less than high school)	NA	0.45	NA	0.04	NA	0.19
High school	0.33 (-1.27,1.94)	NA	0.79 (-1.52, 3.10)	NA	-0.03 (-1.46, 1.40)	NA
Some college	0.28 (-1.39,1.96)	NA	0.53 (-1.90, 2.96)	NA	-0.67 (-2.16, 0.82)	NA
College or more	1.13 (-0.53,2.79)	NA	2.81 (0.43, 5.18)	NA	0.63 (-0.85, 2.10)	NA
Difference in initial post-stroke cognitive score by age (per 10-y increase)	-1.02 (-2.09,0.05)	0.06	-0.97 (-2.33, 0.39)	0.16	-1.54 (-2.52, -0.57)	0.001
Difference in initial post-stroke cognitive score for female sex/gender	0.48 (-0.21,1.18)	0.17	0.47 (-0.45, 1.39)	0.31	0.53 (-0.10, 1.15)	0.09
Post-stroke cognitive slope in those with less than high school education, per y	-0.33 (-0.56, -0.11)	0.003	-0.12 (-0.42, 0.17)	0.40	-0.23 (-0.47, -0.00)	0.047
Difference in post-stroke cognitive slope by education, per y (Reference: less than high school)	NA	0.09	NA	0.02	NA	0.03
High school	0.07 (-0.19, 0.32)	NA	-0.19 (-0.53, 0.15)	NA	0.03 (-0.23, 0.30)	NA
Some college	0.23 (-0.03, 0.49)	NA	-0.17 (-0.54, 0.20)	NA	0.28 (0.02, 0.55)	NA
College or more	-0.01 (-0.26,0.23)	NA	-0.48 (-0.81, -0.14)	NA	0.03 (-0.23, 0.29)	NA
Difference in post-stroke cognitive slope by age (per 10-y increase), per y	-0.25 (-0.46, -0.04)	0.02	-0.13 (-0.37, 0.12)	0.31	0.12 (-0.10, 0.34)	0.29
Difference in post-stroke cognitive slope for female sex/gender, per y	-0.13 (-0.25, 0.00)	0.05	-0.11 (-0.28, 0.06)	0.21	-0.12 (-0.24, 0.01)	0.07
Difference in post-stroke cognitive slope by age (per 10-y increase) and education, per y (Reference: less than high school)	NA	0.66	NA	0.18	NA	0.16
High school	0.08 (-0.18, 0.34)	NA	0.15 (-0.16, 0.46)	NA	-0.17 (-0.43, 0.10)	NA
Some college	-0.05 (-0.31, 0.21)	NA	-0.17 (-0.50, 0.16)	NA	-0.15 (-0.41, 0.12)	NA
College or more	0.03 (-0.21, 0.28)	NA	0.03 (-0.28, 0.33)	NA	-0.28 (-0.53, -0.03)	NA

Abbreviations: CI, confidence interval; y, year. [a]All cognitive measurements are set to a t score metric (mean [SD], 50 [10]). A 1-point difference represents a 0.1-SD difference in the distribution of cognition across the 4 cohorts. Higher cognitive scores indicate better performance. Model includes follow-up time, education, and education x follow-up time, cohort, age, age x follow-up time, education x age, education x age x follow-up time, sex, sex x follow-up time, race, income, pre-stroke mean systolic blood pressure, pre-stroke mean fasting glucose, pre-stroke mean low-density lipoprotein cholesterol, body mass index, smoking status, physical activity, history of myocardial infarction, history of atrial fibrillation, estimated glomerular filtration rate, and stroke type.

© 2025 Springer MV et al. *JAMA Network Open*.

eTable 2. Association of education and post-stroke cognitive decline including adjustment for ApoE genotype among those with genetic data.						
Coefficient	Global Cognition[a]		Executive Function		Memory	
	Estimate (95% CI)	*P*	Estimate (95% CI)	*P*	Estimate (95% CI)	*P*
**Model 1: No adjustment[b]**						
Participants, total No.	1406		1059		1242	
Initial post-stroke cognitive score in those with less than high school education	46.27 (45.26, 47.28)	<0.001	45.46 (44.24, 46.68)	<0.001	48.68 (47.69, 49.66)	<0.001
Difference in initial post-stroke cognitive score by Education (Reference: less than high school)	NA	0.12	NA	0.02	NA	0.70
High school	0.31 (-0.98, 1.61)	NA	0.03 (-1.50, 1.55)	NA	0.30 (-0.94, 1.55)	NA
Some college	0.45 (-0.88, 1.79)	NA	1.03 (-0.57, 2.63)	NA	0.07 (-1.21, 1.36)	NA
College or more	1.41 (0.07, 2.76)	NA	2.00 (0.38, 3.63)	NA	0.64 (-0.64, 1.91)	NA
Post-stroke cognitive slope in those with less than high school education, per y	-0.63 (-0.82, -0.45)	<0.001	-0.38 (-0.59, -0.16)	<0.001	-0.33 (-0.53, -0.14)	<0.001
Difference in post-stroke cognitive slope by education, per y (Reference: less than high school)	NA	0.05	NA	0.08	NA	<0.001
High school	0.10 (-0.14, 0.33)	NA	-0.08 (-0.35, 0.19)	NA	-0.18 (-0.43, 0.06)	NA
Some college	0.29 (0.05, 0.52)	NA	-0.14 (-0.43, 0.15)	NA	0.21 (-0.04, 0.45)	NA
College or more	0.04 (-0.19, 0.27)	NA	-0.33 (-0.60, -0.06)	NA	-0.14 (-0.38, 0.10)	NA
**Model 2: Full adjustment[c]**						
Participants, total No.	1347		1033		1184	
Initial post-stroke cognitive score in those with less than high school education	45.58 (41.76, 49.39)	<0.001	42.18 (37.49, 46.88)	<0.001	50.06 (46.41, 53.70)	<0.001
Difference in initial post-stroke cognitive score by education (Reference: less than high school)	NA	0.47	NA	0.11	NA	0.11
High school	0.35 (-0.95, 1.65)	NA	0.29 (-1.22, 1.79)	NA	0.54 (-0.71, 1.78)	NA
Some college	-0.01 (-1.36, 1.34)	NA	0.16 (-1.44, 1.76)	NA	-0.14 (-1.43, 1.16)	NA
College or more	0.85 (-0.57, 2.26)	NA	1.69 (0.01, 3.37)	NA	1.14 (-0.20, 2.49)	NA
Difference in initial post-stroke cognitive score by age (per 10-y increase)	-1.49 (-2.04, -0.93)	<0.001	-1.47 (-2.15, -0.80)	<0.001	-1.38 (-1.91, -0.85)	<0.001

© 2025 Springer MV et al. *JAMA Network Open*.

Difference in intial post-stroke cognitive score for female sex/gender	0.55 (-0.32, 1.41)	0.21	0.72 (-0.32, 1.76)	0.17	0.68 (-0.15, 1.51)	0.10
Difference in initial post-stroke cognitive score in those with one or more ApoE e4 alleles	0.29 (-0.63, 1.22)	0.52	0.78 (-0.38, 1.93)	0.18	0.02 (-0.87, 0.91)	0.96
Post-stroke cognitive slope in those with less than high school education, per y	-0.26 (-0.47, -0.05)	0.013	-0.12 (-0.37, 0.12)	0.32	-0.10 (-0.32, 0.12)	0.36
Difference in post-stroke cognitive slope by education, per y (Reference: less than high school)	NA	0.02	NA	0.047	NA	0.002
High school	0.06 (-0.16, 0.28)	NA	-0.11 (-0.37, 0.15)	NA	-0.15 (-0.39, 0.09)	NA
Some college	0.26 (0.03, 0.49)	NA	-0.17 (-0.45, 0.11)	NA	0.19 (-0.05, 0.43)	NA
College or more	-0.02 (-0.24, 0.20)	NA	-0.36 (-0.63, -0.09)	NA	-0.16 (-0.39, 0.08)	NA
Difference in post-stroke cognitive slope by age (per 10-y increase), per y	-0.27 (-0.36, -0.19)	<0.001	-0.11 (-0.21, -0.00)	0.046	-0.09 (-0.18, -0.00)	0.04
Difference in post-stroke cognitive slope for female sex/gender, per y	-0.12 (-0.26, 0.02)	0.09	-0.14 (-0.32, 0.04)	0.12	-0.10 (-0.25, 0.04)	0.16
Difference in post-stroke cognitive slope in those with one or more ApoE e4 alleles, per y	-0.26 (-0.42, -0.11)	<0.001	-0.20 (-0.40, 0.01)	0.05	-0.09 (-0.25, 0.07)	0.27

Abbreviations: ApoE e4, apolipoprotein E epsilon 4; CI, confidence interval; y, year. [a]All cognitive measurements are set to a t score metric (mean [SD], 50 [10]). A 1-point difference represents a 0.1-SD difference in the distribution of cognition across the 4 cohorts. Higher cognitive scores indicate better performance. [b]Model 1 includes follow-up time, education, education x follow-up time, and pre-stroke cognition. [c]Model 2 added cohort, age, age x follow- up time, sex, sex x follow-up time, race, income, pre-stroke mean systolic blood pressure, pre-stroke mean fasting glucose, pre-stroke mean low-density lipoprotein cholesterol, body mass index, smoking status, physical activity, history of myocardial infarction, history of atrial fibrillation, estimated glomerular filtration rate, stroke type, ApoE genotype, and ApoE genotype*time to Model 1.

© 2025 Springer MV et al. *JAMA Network Open*.

eTable 3. Association of education and post-stroke cognitive decline, in participants with no history of stroke at baseline.

Coefficient	Global Cognition[a]		Executive Function		Memory	
	Estimate (95% CI)	*P*	Estimate (95% CI)	*P*	Estimate (95% CI)	
**Model 1: No adjustment[b]**						
Participants, total No.	1881		1397		1676	
Initial post-stroke cognitive score in those with less than high school education	46.82 (45.94, 47.69)	<0.001	45.61 (44.47, 46.75)	<0.001	49.01 (48.19, 49.84)	<0.001
Difference in initial post-stroke cognitive score by Education (Reference: less than high school)	NA	0.19	NA	<0.001	NA	0.32
High school	0.05 (-1.05, 1.15)	N/A	-0.50 (-1.90, 0.90)	N/A	0.16 (-0.86, 1.18)	N/A
Some college	0.36 (-0.79, 1.50)	N/A	1.31 (-0.15, 2.78)	N/A	0.20 (-0.86, 1.25)	N/A
College or more	0.94 (-0.20, 2.07)	N/A	1.73 (0.26, 3.21)	N/A	0.79 (-0.24, 1.83)	N/A
Post-stroke cognitive slope in those with less than high school education, per y	-0.63 (-0.80, -0.46)	<0.001	-0.33 (-0.54, -0.13)	0.001	-0.28 (-0.46, -0.10)	0.001
Difference in post-stroke cognitive slope by education, per y (Reference: less than high school)	NA	0.21	NA	0.007	NA	0.001
High school	0.11 (-0.11, 0.32)	N/A	-0.05 (-0.31, 0.21)	N/A	-0.14 (-0.35, 0.08)	N/A
Some college	0.23 (0.01, 0.44)	N/A	-0.28 (-0.56, -0.01)	N/A	0.17 (-0.05, 0.40)	N/A
College or more	0.10 (-0.11, 0.31)	N/A	-0.37 (-0.63, -0.11)	N/A	-0.14 (-0.35, 0.08)	N/A
**Model 2: Full adjustment[c]**						
Participants, total No.	1774		1346		1575	
Initial post-stroke cognitive score in those with less than high school education	47.84 (44.50, 51.18)	<0.001	43.39 (39.30, 47.47)	<0.001	50.15 (47.12, 53.18)	<0.001
Difference in initial post-stroke cognitive score by education (Reference: less than high school)	NA	0.57	NA	0.02	NA	0.05

© 2025 Springer MV et al. *JAMA Network Open*.

High school	-0.09 (-1.20, 1.02)	NA	-0.17 (-1.58, 1.24)	NA	0.17 (-0.84, 1.18)	NA
Some college	-0.05 (-1.22, 1.12)	NA	0.79 (-0.70, 2.28)	NA	-0.15 (-1.21, 0.91)	NA
College or more	0.52 (-0.68, 1.72)	NA	1.74 (0.19, 3.28)	NA	0.99 (-0.09, 2.08)	NA
Difference in initial post-stroke cognitive score by age (per 10-y increase)	-1.45 (-1.91, -0.99)	<0.001	-1.40 (-2.02, -0.79)	<0.001	-1.14 (-1.56, -0.72)	<0.001
Difference in initial post-stroke cognitive score for female sex/gender	0.44 (-0.28, 1.15)	0.23	0.62 (-0.32, 1.56)	0.19	0.44 (-0.21, 1.09)	0.18
Post-stroke cognitive slope in those with less than high school education, per y	-0.35 (-0.54, -0.16)	<0.001	-0.13 (-0.37, 0.10)	0.26	-0.11 (-0.30, 0.09)	0.29
Difference in post-stroke cognitive slope by education, per y (Reference: less than high school)	NA	0.18	NA	0.002	NA	0.002
High school	0.09 (-0.12, 0.30)	NA	-0.09 (-0.35, 0.17)	NA	-0.10 (-0.32, 0.11)	NA
Some college	0.20 (-0.01, 0.41)	NA	-0.31 (-0.58, -0.03)	NA	0.17 (-0.05, 0.39)	NA
College or more	0.03 (-0.18, 0.23)	NA	-0.43 (-0.69, -0.17)	NA	-0.14 (-0.35, 0.07)	NA
Difference in post-stroke cognitive slope by age (per 10-y increase), per y	-0.23 (-0.30, -0.15)	<0.001	-0.12 (-0.22, -0.01)	0.02	-0.06 (-0.14, 0.01)	0.11
Difference in post-stroke cognitive slope for female sex/gender, per y	-0.14 (-0.27, -0.01)	0.03	-0.11 (-0.28, 0.07)	0.23	-0.11 (-0.25, 0.02)	0.09

Abbreviations: CI, confidence interval; y, year. [a]All cognitive measurements are set to a t score metric (mean [SD], 50 [10]). A 1-point difference represents a 0.1-SD difference in the distribution of cognition across the 4 cohorts. Higher cognitive scores indicate better performance. [b]Model 1 includes follow-up time, education, education x follow-up time, and pre-stroke cognition. [c]Model 2 added cohort, age, age x follow-up time, sex, sex x follow-up time, race, income, pre-stroke mean systolic blood pressure, pre-stroke mean fasting glucose, pre-stroke mean low-density lipoprotein cholesterol, body mass index, smoking status, physical activity, history of myocardial infarction, history of atrial fibrillation, estimated glomerular filtration rate, and stroke type to Model 1.

© 2025 Springer MV et al. *JAMA Network Open*.

eTable 4. Association of education and post-stroke cognitive decline including adjustment for post-stroke depressive symptoms.						
**Coefficient**	**Global Cognition[a]**		**Executive Function**		**Memory**	
	**Estimate (95% CI)**	***P***	**Estimate (95% CI)**	***P***	**Estimate (95% CI)**	***P***
**Model 1: No adjustment[b]**						
Participants, total No.	1134		969		1061	
Initial post-stroke cognitive score in those with less than high school education	47.43 (46.46, 48.40)	<0.001	45.27 (44.05, 46.49)	<0.001	48.63 (47.67, 49.59)	<0.001
Difference in initial post-stroke cognitive score by education (Reference: less than high school)	NA	0.22	NA	0.013	NA	0.29
High school	0.44 (-0.81, 1.69)	NA	0.91 (-0.63, 2.44)	NA	0.58 (-0.65, 1.81)	NA
Some college	0.12 (-1.17, 1.41)	NA	2.08 (0.48, 3.67)	NA	0.27 (-0.97, 1.52)	NA
College or more	1.10 (-0.19, 2.38)	NA	2.36 (0.73, 3.99)	NA	1.08 (-0.15, 2.31)	NA
Post-stroke cognitive slope in those with less than high school education, per y	-0.60 (-0.78, -0.42)	<0.001	-0.44 (-0.65, -0.23)	<0.001	-0.28 (-0.47, -0.10)	0.002
Difference in post-stroke cognitive slope by education, per y (Reference: less than high school)	NA	0.07	NA	0.04	NA	<0.001
High school	0.05 (-0.17, 0.27)	NA	0.01 (-0.26, 0.27)	NA	-0.17 (-0.39, 0.06)	NA
Some college	0.24 (0.02, 0.47)	NA	-0.19 (-0.48, 0.09)	NA	0.21 (-0.02, 0.43)	NA
College or more	0.04 (-0.17, 0.26)	NA	-0.30 (-0.57, -0.02)	NA	-0.14 (-0.36, 0.08)	NA
**Model 2: Full adjustment[c]**						
Participants, total No.	1076		932		1005	
Initial post-stroke cognitive score in those with less than high school education	49.34 (45.62, 53.06)	<0.001	44.01 (39.36, 48.65)	<0.001	48.97 (45.45, 52.50)	<0.001
Difference in initial post-stroke cognitive score by education (Reference: less than high school)	NA	0.32	NA	0.10	NA	0.012
High school	0.58 (-0.66, 1.81)	NA	1.03 (-0.48, 2.53)	NA	1.01 (-0.19, 2.20)	NA
Some college	-0.15 (-1.43, 1.14)	NA	1.09 (-0.50, 2.67)	NA	-0.10 (-1.33, 1.13)	NA
College or more	0.73 (-0.61, 2.07)	NA	2.09 (0.42, 3.77)	NA	1.43 (0.16, 2.70)	NA
Difference in initial post-stroke cognitive score by age (per 10-y increase)	-1.52 (-2.08, -0.96)	<0.001	-1.80 (-2.51, -1.08)	<0.001	-1.15 (-1.68, -0.61)	<0.001
Difference in initial post-stroke cognitive score for female sex/gender	0.43 (-0.39, 1.25)	0.30	0.84 (-0.20, 1.89)	0.11	0.99 (0.20, 1.78)	0.013
Difference in initial post-stroke cognitive score by CES- D score (per 1 unit increase)	0.10 (-0.94, 1.13)	0.85	-0.39 (-1.77, 0.98)	0.57	0.05 (-0.94, 1.04)	0.92
Post-stroke cognitive slope in those with less than high school education, per y	-0.03 (-0.28, 0.22)	0.80	-0.45 (-0.81, -0.09)	0.014	0.09 (-0.16, 0.35)	0.47

© 2025 Springer MV et al. *JAMA Network Open*.

Difference in post-stroke cognitive slope by education, per y (Reference: less than high school)	NA	0.08	NA	0.047	NA	0.001
High school	-0.01 (-0.23, 0.20)	NA	-0.03 (-0.29, 0.24)	NA	-0.17 (-0.40, 0.05)	NA
Some college	0.11 (-0.11, 0.33)	NA	-0.17 (-0.46, 0.11)	NA	0.13 (-0.10, 0.36)	NA
College or more	-0.13 (-0.34, 0.09)	NA	-0.32 (-0.60, -0.05)	NA	-0.20 (-0.42, 0.02)	NA
Difference in post-stroke cognitive slope by age (per 10-y increase), per y	-0.22 (-0.30, -0.14)	<0.001	-0.08 (-0.19, 0.02)	0.12	-0.05 (-0.13, 0.04)	0.26
Difference in post-stroke cognitive slope for female sex/gender, per y	-0.11 (-0.25, 0.02)	0.09	-0.14 (-0.32, 0.04)	0.12	-0.14 (-0.28, -0.00)	0.045
Difference in post-stroke cognitive slope by CES-D score (per 1 unit increase), per y	-0.25 (-0.36, -0.13)	<0.001	0.13 (-0.05, 0.30)	0.14	-0.16 (-0.29, -0.04)	0.009

Abbreviations: CES-D, Centers for Epidemiologic Studies Depression; CI, confidence interval; y, year. [a]All cognitive measurements are set to a t score metric (mean [SD], 50 [10]). A 1-point difference represents a 0.1-SD difference in the distribution of cognition across the 4 cohorts. Higher cognitive scores indicate better performance. [b]Model 1 includes follow-up time, education, education x follow-up time, and pre-stroke cognition. [c]Model 2 added cohort, age, age x follow-up time, sex, sex x follow-up time, race, income, pre-stroke mean systolic blood pressure, pre-stroke mean fasting glucose, pre-stroke mean low-density lipoprotein cholesterol, body mass index, smoking status, physical activity, history of myocardial infarction, history of atrial fibrillation, estimated glomerular filtration rate, stroke type, CES-D score, and CES-D score*time to Model 1.

© 2025 Springer MV et al. *JAMA Network Open*.

eTable 5. Association of education and post-stroke decline in global cognitive performance, censoring observations at time of second incident stroke.

Coefficient	Global Cognition[a]		Executive Function		Memory	
	Estimate (95% CI)	*P*	Estimate (95% CI)	*P*	Estimate (95% CI)	*P*
**Model 1: No adjustment[b]**						
Participants, total No.	1944		1377		1732	
Initial post-stroke cognitive score in those with less than high school education	46.99 (46.15, 47.82)	<0.001	45.69 (44.56, 46.82)	<0.001	49.03 (48.24, 49.81)	<0.001
Difference in initial post-stroke cognitive score by education (Reference: less than high school)	NA	0.27	NA	0.001	NA	0.14
High school	0.04 (-1.02, 1.10)	NA	-0.48 (-1.87, 0.91)	NA	0.32 (-0.65, 1.28)	NA
Some college	0.19 (-0.91, 1.29)	NA	1.39 (-0.06, 2.85)	NA	0.34 (-0.66, 1.33)	NA
College or more	0.82 (-0.27, 1.91)	NA	1.59 (0.14, 3.04)	NA	1.02 (0.04, 2.00)	NA
Post-stroke cognitive slope in those with less than high school education, per y	-0.60 (-0.77, -0.42)	<0.001	-0.33 (-0.55, -0.12)	0.002	-0.23 (-0.41, -0.05)	0.011
Difference in post-stroke cognitive slope by education, per y (Reference: less than high school)	NA	0.049	NA	0.02	NA	<0.001
High school	0.15 (-0.07, 0.36)	NA	-0.02 (-0.30, 0.25)	NA	-0.15 (-0.37, 0.07)	NA
Some college	0.29 (0.07, 0.51)	NA	-0.29 (-0.58, 0.00)	NA	0.14 (-0.08, 0.37)	NA
College or more	0.10 (-0.11, 0.30)	NA	-0.30 (-0.57, -0.03)	NA	-0.20 (-0.41, 0.01)	NA
**Model 2: Full adjustment[c]**						
Participants, total No.	1822		1324		1618	
Initial post-stroke cognitive score in those with less than high school education	47.31 (44.05, 50.56)	<0.001	42.72 (38.69, 46.75)	<0.001	49.12 (46.21, 52.04)	<0.001
Difference in initial post-stroke cognitive score by Education (Reference: less than high school)	NA	0.68	NA	0.03	NA	0.015
High school	-0.06 (-1.14, 1.02)	NA	-0.07 (-1.48, 1.33)	NA	0.41 (-0.56, 1.38)	NA
Some college	-0.23 (-1.36, 0.90)	NA	0.98 (-0.50, 2.47)	NA	-0.01 (-1.03, 1.00)	NA
College or more	0.33 (-0.83, 1.49)	NA	1.65 (0.12, 3.18)	NA	1.26 (0.22, 2.30)	NA
Difference in initial post-stroke cognitive score by age (per 10-y increase)	-1.40 (-1.85, -0.95)	<0.001	-1.38 (-1.98, -0.77)	<0.001	-1.01 (-1.41, -0.61)	<0.001

© 2025 Springer MV et al. *JAMA Network Open*.

Difference in initial post-stroke cognitive score for female sex/gender	0.49 (-0.21, 1.19)	0.16	0.54 (-0.39, 1.48)	0.25	0.58 (-0.05, 1.21)	0.06
Post-stroke cognitive slope in those with less than high school education, per y	-0.35 (-0.55, -0.15)	<0.001	-0.11 (-0.37, 0.14)	0.38	-0.02 (-0.22, 0.18)	0.81
Difference in post-stroke cognitive slope by education, per y (Reference: less than high school)	NA	0.09	NA	0.011	NA	0.001
High school	0.12 (-0.09, 0.34)	NA	-0.09 (-0.37, 0.19)	NA	-0.14 (-0.36, 0.08)	NA
Some college	0.23 (0.02, 0.45)	NA	-0.33 (-0.62, -0.04)	NA	0.13 (-0.09, 0.35)	NA
College or more	0.04 (-0.17, 0.25)	NA	-0.38 (-0.66, -0.11)	NA	-0.20 (-0.41, 0.01)	NA
Difference in post-stroke cognitive slope by age (per 10-y increase), per y	-0.21 (-0.29, -0.13)	<0.001	-0.12 (-0.22, -0.01)	0.03	-0.07 (-0.15, 0.01)	0.08
Difference in post-stroke cognitive slope for female sex/gender, per y	-0.12 (-0.25, 0.02)	0.08	-0.13 (-0.31, 0.06)	0.17	-0.15 (-0.28, -0.02)	0.02

Abbreviations: CI, confidence interval; y, year. [a]All cognitive measurements are set to a t score metric (mean [SD], 50 [10]). A 1-point difference represents a 0.1-SD difference in the distribution of cognition across the 4 cohorts. Higher cognitive scores indicate better performance. [b]Model 1 includes follow-up time, education, education x follow-up time, and pre-stroke cognition. [c]Model 2 added cohort, age, age x follow-up time, sex, sex x follow-up time, race, income, pre-stroke mean systolic blood pressure, pre-stroke mean fasting glucose, pre-stroke mean low-density lipoprotein cholesterol, body mass index, smoking status, physical activity, history of myocardial infarction, history of atrial fibrillation, estimated glomerular filtration rate, and stroke type to Model 1.

© 2025 Springer MV et al. *JAMA Network Open*.

eTable 6. Association of education and post-stroke cognitive decline, in participants of ARIC.

Coefficient	Global Cognition[a]		Executive Function		Memory	
	Estimate (95% CI)	*P*	Estimate (95% CI)	*P*	Estimate (95% CI)	*P*
**Model 1: No adjustment[b]**						
Participants, total No.	282		280		249	
Initial post-stroke cognitive score in those with less than high school education	40.43 (36.89, 43.97)	<0.001	40.35 (37.37, 43.33)	<0.001	42.87 (38.50, 47.24)	<0.001
Difference in initial post-stroke cognitive score by education (Reference: less than high school)	NA	0.05	NA	0.19	NA	0.22
High school	3.37 (-0.99, 7.72)	NA	2.45 (-1.24, 6.14)	NA	-0.85 (-6.30, 4.60)	NA
Some college	1.13 (-4.42, 6.68)	NA	2.62 (-2.16, 7.40)	NA	-3.30 (-10.46, 3.85)	NA
College or more	5.75 (1.27, 10.22)	NA	4.14 (0.36, 7.91)	NA	2.76 (-2.68, 8.20)	NA
Post-stroke cognitive slope in those with less than high school education, per y	-0.26 (-0.66, 0.14)	0.20	0.03 (-0.35, 0.40)	0.88	-0.64 (-1.18, -0.10)	0.02
Difference in post-stroke cognitive slope by education, per y (Reference: less than high school)	NA	0.25	NA	0.13	NA	0.18
High school	-0.11 (-0.60, 0.39)	NA	-0.20 (-0.67, 0.28)	NA	0.21 (-0.47, 0.89)	NA
Some college	0.26 (-0.43, 0.95)	NA	-0.37 (-1.04, 0.30)	NA	0.73 (-0.23, 1.70)	NA
College or more	-0.31 (-0.80, 0.17)	NA	-0.52 (-0.98, -0.06)	NA	-0.18 (-0.84, 0.48)	NA
**Model 2: Full adjustment[c]**						
Participants, total No.	281		279		248	
Initial post-stroke cognitive score in those with less than high school education	32.50 (20.67, 44.33)	<0.001	35.32 (24.83, 45.81)	<0.001	49.73 (34.87, 64.59)	<0.001
Difference in initial post-stroke cognitive score by education (Reference: less than high school)	NA	0.045	NA	0.36	NA	0.25
High school	3.21 (-1.09, 7.52)	NA	2.25 (-1.58, 6.07)	NA	-1.65 (-7.34, 4.03)	NA

© 2025 Springer MV et al. *JAMA Network Open*.

Some college	-0.88 (-6.31, 4.55)	NA	0.94 (-3.93, 5.80)	NA	-5.21 (-12.47, 2.05)	NA
College or more	5.05 (0.55, 9.54)	NA	3.37 (-0.58, 7.32)	NA	1.15 (-4.57, 6.88)	NA
Difference in initial post-stroke cognitive score by age (per 10-y increase)	-1.53 (-3.10, 0.04)	0.05	-0.87 (-2.27, 0.52)	0.22	-4.35 (-6.47, -2.23)	<0.001
Difference in initial post-stroke cognitive score for female sex/gender	1.20 (-1.81, 4.21)	0.43	2.14 (-0.53, 4.81)	0.11	2.20 (-1.78, 6.18)	0.27
Post-stroke cognitive slope in those with less than high school education, per y	-0.26 (-0.66, 0.13)	0.19	0.06 (-0.34, 0.45)	0.77	-0.66 (-1.25, -0.07)	0.02
Difference in post-stroke cognitive slope by education, per y (Reference: less than high school)	NA	0.36	NA	0.18	NA	0.19
High school	-0.11 (-0.58, 0.36)	NA	-0.24 (-0.71, 0.23)	NA	0.23 (-0.46, 0.92)	NA
Some college	0.26 (-0.40, 0.92)	NA	-0.35 (-1.03, 0.32)	NA	0.81 (-0.17, 1.79)	NA
College or more	-0.24 (-0.69, 0.21)	NA	-0.50 (-0.95, -0.04)	NA	-0.13 (-0.79, 0.54)	NA
Difference in post-stroke cognitive slope by age (per 10-y increase), per y	-0.26 (-0.46, -0.06)	0.010	-0.17 (-0.38, 0.03)	0.09	0.10 (-0.19, 0.39)	0.48
Difference in post-stroke cognitive slope for female sex/gender, per y	-0.18 (-0.51, 0.15)	0.28	-0.22 (-0.55, 0.11)	0.19	-0.28 (-0.76, 0.20)	0.24

Abbreviations: CI, confidence interval; y, year. [a]All cognitive measurements are set to a t score metric (mean [SD], 50 [10]). A 1-point difference represents a 0.1-SD difference in the distribution of cognition across the 4 cohorts. Higher cognitive scores indicate better performance. [b]Model 1 includes follow-up time, education, education x follow-up time, and pre-stroke cognition. [c]Model 2 added age, age x follow-up time, sex, sex x follow-up time, race, income, pre-stroke mean systolic blood pressure, pre-stroke mean fasting glucose, pre-stroke mean low-density lipoprotein cholesterol, body mass index, smoking status, physical activity, history of myocardial infarction, history of atrial fibrillation, estimated glomerular filtration rate, and stroke type to Model 1.

© 2025 Springer MV et al. *JAMA Network Open*.

eTable 7. Association of education and post-stroke cognitive decline, in participants of CHS.						
**Coefficient**	**Global Cognition[a]**		**Executive Function**		**Memory**	
	**Estimate (95% CI)**	***P***	**Estimate (95% CI)**	***P***	**Estimate (95% CI)**	***P***
**Model 1: No adjustment[b]**						
Participants, total No.	433		433		303	
Initial post-stroke cognitive score in those with less than high school education	47.69 (46.10, 49.28)	<0.001	47.17 (45.61, 48.73)	<0.001	49.13 (48.02, 50.25)	<0.001
Difference in initial post-stroke cognitive score by education (Reference: less than high school)	NA	0.22	NA	0.79	NA	0.41
High school	-0.99 (-3.13, 1.14)	NA	-0.95 (-2.97, 1.06)	NA	1.11 (-0.48, 2.71)	NA
Some college	-0.89 (-3.18, 1.39)	NA	-0.68 (-2.83, 1.48)	NA	0.85 (-0.85, 2.54)	NA
College or more	-2.61 (-5.08, -0.14)	NA	-0.23 (-2.56, 2.11)	NA	1.43 (-0.43, 3.28)	NA
Post-stroke cognitive slope in those with less than high school education, per y	-1.30 (-1.66, -0.94)	<0.001	-0.52 (-0.84, -0.20)	0.001	-0.12 (-0.45, 0.20)	0.44
Difference in post-stroke cognitive slope by education, per y (Reference: less than high school)	NA	0.86	NA	0.67	NA	0.13
High school	0.15 (-0.35, 0.65)	NA	0.02 (-0.42, 0.46)	NA	-0.18 (-0.64, 0.27)	NA
Some college	0.00 (-0.53, 0.53)	NA	-0.24 (-0.70, 0.22)	NA	0.35 (-0.14, 0.85)	NA
College or more	0.19 (-0.38, 0.76)	NA	-0.10 (-0.60, 0.40)	NA	-0.22 (-0.75, 0.31)	NA
**Model 2: Full adjustment[c]**						
Participants, total No.	424		424		296	
Initial post-stroke cognitive score in those with less than high school education	49.87 (42.11, 57.63)	<0.001	46.61 (39.03, 54.18)	<0.001	46.28 (40.70, 51.86)	<0.001
Difference in initial post-stroke cognitive score by Education (Reference: less than high school)	NA	0.33	NA	0.86	NA	0.35
High school	-1.07 (-3.24, 1.11)	NA	-0.69 (-2.76, 1.37)	NA	1.08 (-0.53, 2.69)	NA
Some college	-0.96 (-3.30, 1.38)	NA	-0.86 (-3.09, 1.36)	NA	0.56 (-1.18, 2.29)	NA
College or more	-2.49 (-5.15, 0.17)	NA	-0.88 (-3.42, 1.66)	NA	1.65 (-0.34, 3.63)	NA
Difference in initial post-stroke cognitive score by age (per 10-y increase)	-2.65 (-3.99, -1.31)	<0.001	-0.37 (-1.66, 0.91)	0.56	-1.67 (-2.84, -0.51)	0.004
Difference in initial post-stroke cognitive score for female sex/gender	-0.04 (-1.75, 1.67)	0.96	0.52 (-1.12, 2.16)	0.53	1.35 (0.02, 2.67)	0.046
Post-stroke cognitive slope in those with less than high school education, per y	-0.79 (-1.41, -0.18)	0.010	-0.38 (-0.91, 0.15)	0.15	-0.03 (-0.57, 0.51)	0.90
Difference in post-stroke cognitive slope by education, per y (Reference: less than high school)	NA	0.95	NA	0.61	NA	0.09
High school	0.13 (-0.38, 0.64)	NA	0.01 (-0.43, 0.45)	NA	-0.19 (-0.67, 0.28)	NA

© 2025 Springer MV et al. *JAMA Network Open*.

Some college	0.01 (-0.52, 0.55)	NA	-0.25 (-0.71, 0.22)	NA	0.40 (-0.11, 0.91)	NA
College or more	0.04 (-0.54, 0.62)	NA	-0.18 (-0.68, 0.33)	NA	-0.26 (-0.81, 0.29)	NA
Difference in post-stroke cognitive slope by age (per 10-y increase), per y	-0.31 (-0.66, 0.04)	0.08	-0.01 (-0.32, 0.29)	0.94	0.01 (-0.33, 0.35)	0.95
Difference in post-stroke cognitive slope for female sex/gender, per y	-0.15 (-0.55, 0.25)	0.46	-0.14 (-0.49, 0.20)	0.41	-0.26 (-0.64, 0.11)	0.17

Abbreviations: CI, confidence interval; y, year. [a]All cognitive measurements are set to a t score metric (mean [SD], 50 [10]). A 1-point difference represents a 0.1-SD difference in the distribution of cognition across the 4 cohorts. Higher cognitive scores indicate better performance. [b]Model 1 includes follow-up time, education, education x follow-up time, and pre-stroke cognition. [c]Model 2 added age, age x follow-up time, sex, sex x follow-up time, race, income, pre-stroke mean systolic blood pressure, pre-stroke mean fasting glucose, pre-stroke mean low-density lipoprotein cholesterol, body mass index, smoking status, physical activity, history of myocardial infarction, history of atrial fibrillation, estimated glomerular filtration rate, and stroke type to Model 1.

© 2025 Springer MV et al. *JAMA Network Open*.

eTable 8. Association of education and post-stroke cognitive decline, in participants of REGARDS.						
**Coefficient**	**Global Cognition[a]**		**Executive Function**		**Memory**	
	**Estimate (95% CI)**	***P***	**Estimate (95% CI)**	***P***	**Estimate (95% CI)**	***P***
**Model 1: No adjustment[b]**						
Participants, total No.	1169		604		1119	
Initial post-stroke cognitive score in those with less than high school education	48.07 (46.99, 49.15)	<0.001	44.28 (42.04, 46.53)	<0.001	51.51 (50.75, 52.28)	<0.001
Difference in initial post-stroke cognitive score by education (Reference: less than high school)		0.017		<0.001		0.049
High school	0.08 (-1.20, 1.35)		0.23 (-2.35, 2.81)		0.44 (-0.44, 1.33)	
Some college	0.86 (-0.43, 2.16)		3.90 (1.31, 6.49)		0.61 (-0.29, 1.51)	
College or more	1.47 (0.20, 2.74)		3.43 (0.87, 5.99)		1.13 (0.25, 2.01)	
Post-stroke cognitive slope in those with less than high school education, per y	-0.37 (-0.62, -0.13)	0.002	-0.14 (-0.65, 0.36)	0.57	-0.04 (-0.21, 0.12)	0.61
Difference in post-stroke cognitive slope by education, per y (Reference: less than high school)		0.08		0.004		0.10
High school	0.18 (-0.11, 0.47)		-0.14 (-0.74, 0.46)		-0.03 (-0.23, 0.16)	
Some college	0.18 (-0.10, 0.47)		-0.72 (-1.30, -0.15)		0.04 (-0.15, 0.24)	
College or more	-0.03 (-0.31, 0.24)		-0.69 (-1.26, -0.12)		-0.13 (-0.32, 0.06)	
**Model 2: Full adjustment[c]**						
Participants, total No.	1050		557		1006	
Initial post-stroke cognitive score in those with less than high school education	50.43 (45.32, 55.53)	<0.001	44.24 (34.80, 53.67)	<0.001	51.14 (47.73, 54.54)	<0.001
Difference in initial post-stroke cognitive score by education (Reference: less than high school)		0.27		<0.001		0.30
High school	-0.18 (-1.53, 1.17)		0.47 (-2.27, 3.21)		0.29 (-0.65, 1.24)	
Some college	0.47 (-0.91, 1.85)		3.77 (0.99, 6.54)		0.31 (-0.66, 1.28)	
College or more	0.81 (-0.59, 2.22)		3.70 (0.89, 6.50)		0.82 (-0.16, 1.79)	
Difference in initial post-stroke cognitive score by age (per 10-y increase)	-1.26 (-1.78, -0.74)	<0.001	-2.10 (-3.14, -1.07)	<0.001	-0.35 (-0.71, 0.01)	0.05
Difference in initial post-stroke cognitive score for female sex/gender	1.17 (0.37, 1.97)	0.004	-0.09 (-1.59, 1.42)	0.91	0.72 (0.16, 1.28)	0.012

© 2025 Springer MV et al. *JAMA Network Open*.

Post-stroke cognitive slope in those with less than high school education, per y	-0.23 (-0.51, 0.05)	0.10	-0.16 (-0.80, 0.47)	0.61	0.11 (-0.08, 0.30)	0.27
Difference in post-stroke cognitive slope by education, per y (Reference: less than high school)		0.08		0.008		0.06
High school	0.13 (-0.17, 0.44)		-0.20 (-0.88, 0.47)		-0.06 (-0.27, 0.15)	
Some college	0.14 (-0.16, 0.43)		-0.75 (-1.40, -0.10)		0.01 (-0.19, 0.22)	
College or more	-0.09 (-0.39, 0.20)		-0.79 (-1.43, -0.15)		-0.17 (-0.37, 0.03)	
Difference in post-stroke cognitive slope by age (per 10-y increase), per y	-0.18 (-0.29, -0.07)	<0.001	-0.06 (-0.29, 0.16)	0.58	-0.13 (-0.21, -0.06)	<0.001
Difference in post-stroke cognitive slope for female sex/gender, per y	-0.00 (-0.17, 0.16)	0.95	0.16 (-0.18, 0.51)	0.35	-0.09 (-0.20, 0.03)	0.12

Abbreviations: CI, confidence interval; y, year. [a]All cognitive measurements are set to a t score metric (mean [SD], 50 [10]). A 1-point difference represents a 0.1-SD difference in the distribution of cognition across the 4 cohorts. Higher cognitive scores indicate better performance. [b]Model 1 includes follow-up time, education, education x follow-up time, and pre-stroke cognition. [c]Model 2 added age, age x follow-up time, sex, sex x follow-up time, race, income, pre-stroke mean systolic blood pressure, pre-stroke mean fasting glucose, pre-stroke mean low-density lipoprotein cholesterol, body mass index, smoking status, physical activity, history of myocardial infarction, history of atrial fibrillation, estimated glomerular filtration rate, and stroke type to Model 1.

© 2025 Springer MV et al. *JAMA Network Open*.

eTable 9. Association of education and post-stroke cognitive decline in which Participants with pre-stroke cognitive function in or below the 5th percentile are dropped.

Coefficient	Global Cognition[1]		Executive Function		Memory	
	Estimate (95% CI)	*P value*	Estimate (95% CI)	*P value*	Estimate (95% CI)	*P value*
**Model 1: No adjustment[2]**						
Participants, total No.	1918		1376		1712	
Initial post-stroke cognitive score in those with less than high school education	46.89 (46.00, 47.77)	<0.001	45.20 (44.01, 46.38)	<0.001	48.99 (48.16, 49.82)	<0.001
Difference in initial post-stroke cognitive score by education		0.11		<0.001		0.12
High school vs less than high school	0.07 (-1.01, 1.15)		-0.06 (-1.49, 1.37)		0.55 (-0.43, 1.53)	
Some college vs less than high school	0.44 (-0.67, 1.56)		1.75 (0.26, 3.24)		0.59 (-0.42, 1.60)	
College or more vs less than high school	1.04 (-0.06, 2.14)		2.19 (0.70, 3.67)		1.16 (0.18, 2.15)	
Post-stroke cognitive slope in those with less than high school education, per y	- 0.63 (-0.81, -0.45)	<0.001	-0.36 (-0.57, -0.15)	<0.001	-0.28 (-0.46, -0.10)	0.002
Difference in post-stroke cognitive slope by education, per y		0.10		0.011		<0.001
High school vs less than high school	0.14 (-0.08, 0.36)		-0.03 (-0.30, 0.23)		-0.13 (-0.35, 0.09)	
Some college vs less than high school	0.26 (0.04, 0.48)		-0.24 (-0.52, 0.03)		0.17 (-0.05, 0.40)	
College or more vs less than high school	0.10 (-0.11, 0.31)		-0.34 (-0.61, -0.08)		-0.15 (-0.37, 0.06)	
**Model 2: Full adjustment[3]**						

© 2025 Springer MV et al. *JAMA Network Open*.

Participants, total No.	1795		1320		1597	
Initial post-stroke cognitive score in those with less than high school education	48.85 (45.29, 52.41)	<0.001	44.58 (40.33, 48.84)	<0.001	50.22 (47.17, 53.27)	<0.001
Difference in initial post-stroke cognitive score by education		0.60		0.003		0.04
High school vs less than high school	0.03 (-1.08, 1.14)		0.34 (-1.10, 1.78)		0.50 (-0.50, 1.49)	
Some college vs less than high school	-0.02 (-1.19, 1.14)		1.32 (-0.20, 2.83)		0.10 (-0.94, 1.14)	
College or more vs less than high school	0.54 (-0.65, 1.73)		2.38 (0.82, 3.94)		1.18 (0.12, 2.24)	
Difference in initial post-stroke cognitive score by age (per 10-y increase)	-1.31 (-1.77, -0.86)	<0.001	-1.55 (-2.17, -0.94)	<0.001	-0.99 (-1.40, -0.59)	<0.001
Difference in initial post-stroke cognitive score for female sex/gender	0.65 (-0.05, 1.35)	0.07	0.39 (-0.54, 1.32)	0.41	0.51 (-0.12, 1.15)	0.11
Post-stroke cognitive slope in those with less than high school education, per y	-0.36 (-0.56, -0.15)	<0.001	-0.21 (-0.46, 0.04)	0.10	-0.12 (-0.32, 0.08)	0.22
Difference in post-stroke cognitive slope by education, per y		0.10		0.005		0.001
High school vs less than high school	0.11 (-0.11, 0.32)		-0.05 (-0.32, 0.21)		-0.08 (-0.30, 0.14)	
Some college vs less than high school	0.21 (-0.01, 0.43)		-0.25 (-0.53, 0.03)		0.18 (-0.04, 0.40)	
College or more vs less than high school	0.02 (-0.19, 0.23)		-0.39 (-0.66, -0.12)		-0.15 (-0.36, 0.07)	
Difference in post-stroke cognitive slope by age (per 10-y increase), per y	-0.22 (-0.30, -0.14)	<0.001	-0.09 (-0.19, 0.01)	0.09	-0.07 (-0.15, 0.01)	0.06
Difference in post-stroke cognitive slope for female sex/gender, per y	-0.13 (-0.26, 0.00)	0.05	-0.10 (-0.27, 0.07)	0.26	-0.11 (-0.24, 0.02)	0.10

Abbreviations: CI, confidence interval; y, year. [1]All cognitive measurements are set to a t score metric (mean [SD], 50 [10]). A 1-point difference represents a 0.1-SD difference in the distribution of cognition across the 4 cohorts. Higher cognitive scores indicate better performance. [2]Model 1 includes follow-up time, education, and education x follow-up time. [3]Model 2 added cohort, age, age x follow-up time, sex, sex x follow-up time, race, income, pre-stroke mean systolic blood pressure, pre-stroke mean fasting glucose, pre-stroke mean low-density lipoprotein cholesterol, body mass index, smoking status, physical activity, history of myocardial infarction, history of atrial fibrillation, estimated glomerular filtration rate, and stroke type to Model 1.

© 2025 Springer MV et al. *JAMA Network Open*.

eTable 10. Association of education and post-stroke cognitive decline in a joint model of longitudinal and survival data.						
**Coefficient**	**Global Cognition[1]**		**Executive Function**		**Memory**	
	**Estimate (95% CI)**	***P value***	**Estimate (95% CI)**	***P value***	**Estimate (95% CI)**	***P value***
**Model 1: No adjustment[2]**						
Participants, total No.	2019		1451		1805	
Initial post-stroke cognitive score in those with less than high school education	45.83 (44.99, 46.66)	<0.001	44.92 (43.90, 45.95)	<0.001	48.32 (47.52, 49.13)	<0.001
Difference in initial post-stroke cognitive score by education		0.003		0.009		<0.001
High school vs less than high school	0.51 (-0.55, 1.57)		-0.32 (-1.57, 0.94)		0.35 (-0.64, 1.35)	
Some college vs less than high school	0.96 (-0.15, 2.08)		1.15 (-0.18, 2.48)		0.77 (-0.27, 1.81)	
College or more vs less than high school	1.18 (0.08, 2.28)		1.06 (-0.26, 2.39)		0.81 (-0.21, 1.82)	
Post-stroke cognitive slope in those with less than high school education, per y	-0.41 (-0.54, -0.28)	<0.001	-0.22 (-0.35, -0.10)	<0.001	-0.11 (-0.22, 0.00)	0.048
Difference in post-stroke cognitive slope by education, per y		0.24		0.28		<0.001
High school vs less than high school	0.05 (-0.11, 0.21)		-0.06 (-0.22, 0.10)		-0.14 (-0.27, -0.01)	
Some college vs less than high school	0.16 (-0.01, 0.32)		-0.12 (-0.30, 0.06)		0.11 (-0.02, 0.25)	
College or more vs less than high school	0.04 (-0.12, 0.20)		-0.15 (-0.31, 0.01)		-0.12 (-0.25, 0.00)	
**Model 2: Full adjustment[3]**						
Participants, total No.	1890		1394		1684	
Initial post-stroke cognitive score in those with less than high school education	47.35 (44.12, 50.57)	<0.001	43.90 (40.03, 47.77)	<0.001	49.44 (46.57, 52.32)	<0.001
Difference in initial post-stroke cognitive score by education		0.09		0.37		0.06
High school vs less than high school	0.40 (-0.65, 1.45)		-0.13 (-1.37, 1.11)		0.38 (-0.52, 1.29)	
Some college vs less than high school	0.36 (-0.75, 1.48)		0.34 (-0.98, 1.67)		0.11 (-0.85, 1.07)	
College or more vs less than high school	0.72 (-0.42, 1.86)		0.91 (-0.46, 2.28)		1.00 (0.03, 1.98)	
Difference in initial post-stroke cognitive score by age (per 10-y increase)	-1.54 (-1.98, -1.10)	<0.001	-1.53 (-2.08, -0.98)	<0.001	-1.16 (-1.54, -0.77)	<0.001
Difference in initial post-stroke cognitive score for female sex/gender	0.38 (-0.30, 1.07)	0.27	0.28 (-0.55, 1.12)	0.51	0.50 (-0.09, 1.09)	0.1
Post-stroke cognitive slope in those with less than high school education, per y	-0.27 (-0.42, -0.12)	<0.001	-0.19 (-0.34, -0.03)	0.02	-0.11 (-0.23, 0.01)	0.07
Difference in post-stroke cognitive slope by education, per y		0.13		0.18		<0.001
High school vs less than high school	0.01 (-0.15, 0.17)		-0.08 (-0.24, 0.08)		-0.11 (-0.24, 0.03)	

© 2025 Springer MV et al. *JAMA Network Open*.

Some college vs less than high school	0.12 (-0.05, 0.29)		-0.11 (-0.29, 0.07)		0.15 (0.01, 0.29)	
College or more vs less than high school	-0.04 (-0.20, 0.12)		-0.18 (-0.34, -0.01)		-0.11 (-0.24, 0.03)	
Difference in post-stroke cognitive slope by age (per 10-y increase), per y	-0.17 (-0.23, -0.11)	<0.001	-0.05 (-0.12, 0.01)	0.09	-0.00 (-0.05, 0.05)	0.92
Difference in post-stroke cognitive slope for female sex/gender, per y	-0.10 (-0.20, 0.00)	0.05	-0.09 (-0.20, 0.02)	0.12	-0.12 (-0.19, -0.03)	0.007

Abbreviations: CI, confidence interval; y, year. [1]All cognitive measurements are set to a t score metric (mean [SD], 50 [10]). A 1-point difference represents a 0.1-SD difference in the distribution of cognition across the 4 cohorts. Higher cognitive scores indicate better performance. [2]Model 1 includes follow-up time, education, and education x follow-up time. [3]Model 2 added cohort, age, age x follow-up time, sex, sex x follow-up time, race, income, pre-stroke mean systolic blood pressure, pre-stroke mean fasting glucose, pre-stroke mean low-density lipoprotein cholesterol, body mass index, smoking status, physical activity, history of myocardial infarction, history of atrial fibrillation, estimated glomerular filtration rate, and stroke type to Model 1. The survival portion of the joint model was included to account for non-random attrition due to death. Results are not displayed.

© 2025 Springer MV et al. *JAMA Network Open*.
