# Supplementary material for: Education Levels and Poststroke Cognitive Trajectories
Source: JAMA Netw Open. 2025 Mar 26;8(3):e252002. doi: 10.1001/jamanetworkopen.2025.2002 (PMC11947833; doi:10.1001/jamanetworkopen.2025.2002)
Supplement: Supplement 2. — Data Sharing Statement [file jamanetwopen-e252002-s002.pdf]

Data Sharing Statement

Springer. Education Levels and Poststroke Cognitive Trajectories. *JAMA Netw Open*.
Published March 26, 2025. doi:10.1001/jamanetworkopen.2025.2002

Data
Data available: Data are available from the corresponding author upon research team and cohort approval and data use agreements with cohorts and the corresponding author's institution.
